# Universal Interatomic Potentials as Configuration-Space Generators for One-Shot and Iterative Fine-Tuning of Ab Initio-Accurate Material-Specific Models

**DOI:** 10.1021/acs.jpclett.6c02067

**Published:** 2026-08-26

**Authors:** Jonas Hänseroth, Aaron Flötotto, Christian Dreßler

**Affiliations:** † Theoretical Solid State Physics, Institute of Physics, Technische Universität Ilmenau, 98693 Ilmenau, Germany

## Abstract

Universal machine learning interatomic potentials (MLIPs) are rapidly becoming general-purpose tools for atomistic simulation, but their role in quantitative materials modeling when reactive events are involved remains unsettled. We compare five universal MLIPs across seven chemically diverse systems and find that strong performance on standard benchmarks does not guarantee accurate predictions of the target observables. In particular, zero-shot models do not reliably reproduce reactive, transport, or high-barrier processes, exemplified here in particular by the sulfur-vacancy jump in MoS_2_. We therefore benchmark a practical alternative against target observables: universal MLIPs are used to generate long molecular dynamics trajectories, the resulting configurations are subsampled and relabeled with DFT, and material-specific MLIPs are subsequently trained or fine-tuned on the resulting first-principles data sets. This workflow converts universal models into efficient configuration-space generators while retaining ab initio reference labels for training and turns their systematic softening of the potential energy surface from a liability into a sampling advantage. Across the tested systems, 2000 DFT-recalculated structures are often sufficient to obtain accurate fine-tuned or trained-from-scratch models. For the most challenging case, iterative self-training progressively refines the sampled configuration space and recovers the DFT MoS_2_ potential energy profile with only 600 first-principles calculations in total. For a 512-atom system on a single nvidia A100 GPU and four 32-core CPU nodes, the complete workflow—including training data generation, DFT labeling, and model creation—delivers 1,000,000 ab initio-quality MD steps in 3 days.

## Graphical abstract

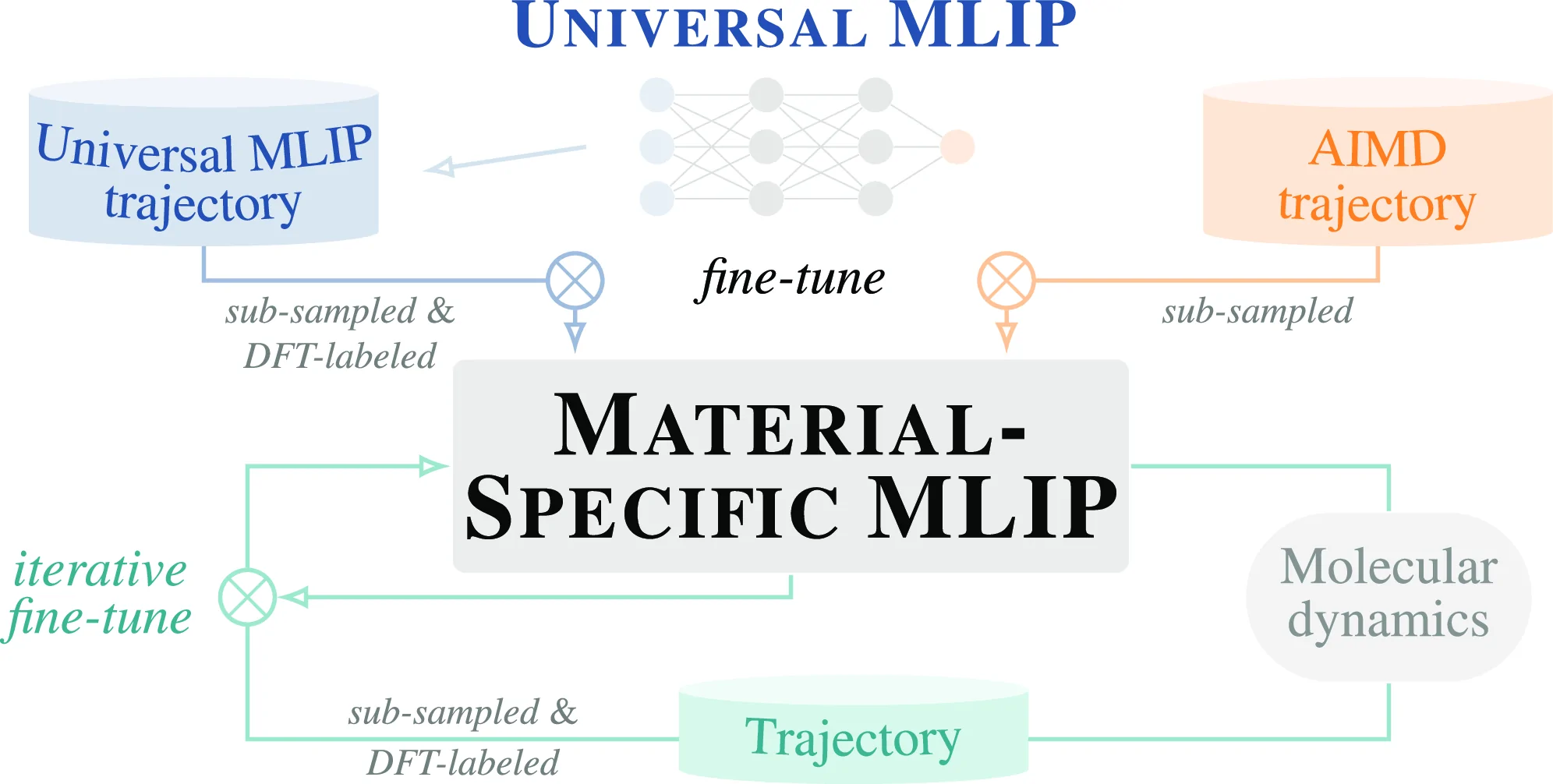

Atomistic simulations are central to understanding and designing functional materials, but they remain limited by the long-standing trade-off between accuracy and computational cost. Ab initio molecular dynamics (AIMD), most commonly based on density functional theory (DFT), provides access to chemical bonding and reaction mechanisms without empirical parametrization, but is typically restricted to comparatively small systems and short time scales.^1–3^ Classical force fields extend simulations to much larger length and time scales, but their accuracy and transferability are limited by the fixed functional form and the domain in which they were parametrized.^4–6^ This accuracy-efficiency dilemma is particularly restrictive for processes such as ion transport, defect migration, hydrogen-bond rearrangements, solvation dynamics, and bond breaking, where long trajectories are needed but the relevant physics is controlled by subtle changes in the potential energy surface.^7–10^

Machine-learning interatomic potentials (MLIPs) have emerged as a powerful route to bridge this gap by learning DFT-quality potential energy surfaces from reference calculations while retaining a computational cost closer to classical molecular dynamics.^11–14^ Early neural-network potentials and Gaussian approximation potentials demonstrated that quantum-mechanical energies and forces can be represented accurately from data.^15–17^ More recently, graph neural networks, equivariant architectures, and symmetry-preserving representations have substantially improved the accuracy, stability, and transferability of MLIPs across chemical environments.^11,18–25^ As a result, MLIPs are increasingly used to obtain nanosecond-scale trajectories with ab initio accuracy for materials and molecular systems that are inaccessible to direct AIMD.

The most recent development in this field is the emergence of universal, or foundation, MLIPs. Instead of training a potential for a single material or chemical system, these models are pretrained on large and chemically diverse databases containing millions of DFT-labeled structures.^26–30^ Universal MLIPs therefore offer the appealing possibility of using a single pretrained model as a general-purpose simulation engine for a broad range of materials.

However, the central question is not only whether universal MLIPs perform well on benchmark metrics but whether they can reproduce the specific observables that motivate a simulation.^31,32^ Many scientifically relevant observables depend on rare or reactive events from the high-barrier regions of configuration space rather than on equilibrium structures alone.^7,33,34^ Examples include diffusion barriers, proton-transfer pathways, and defect migration events.^35^ Recent studies have therefore shown that, despite their broad applicability, universal models often require material-specific adaptation before quantitative accuracy can be expected in specialized applications.^7,34,36–42^

Fine-tuning provides one route to this material-specific accuracy.^43^ By adapting a pretrained model to a smaller DFT-labeled data set of the target system, one can correct the potential energy surface in the region relevant to the material of interest.^7,34,36–40,42,44^

Several specialized fine-tuning strategies can further control how much the pretrained model is adapted. In frozen-layer fine-tuning, only selected parts of the network, often the final interaction or readout layers, are optimized, while the remaining parameters are fixed.^44^ This can reduce the risk of overfitting and preserve the general chemical knowledge of the foundation model but may limit flexibility when the target system differs strongly from the pretraining distribution. Low-rank adaptation (LoRA) approaches instead add a small number of trainable low-rank parameters to the pretrained model, enabling efficient adaptation with reduced memory requirements and fewer trainable weights.^45,46^ Multihead replay fine-tuning keeps a shared representation of the atomic environments while assigning separate output heads to different data sets or tasks.^20,47,48^ During replay, previously learned data sets are included again in the training process, which helps retain earlier information while the shared model is adapted to newly generated configurations.

This study builds on our previous work. In ref 35 we established that fine-tuning renders a range of MLIP architectures equivalent in accuracy when trained on 2000 AIMD-derived configurations, thereby shifting the critical question from the choice of architecture to the choice of training configurations, the question addressed here. References 7, 9, 34, 36, and 49–51 provide the systems studied below, together with the DFT settings and the physical understanding of their respective challenges; they were application studies in which MLIPs served as tools rather than objects of investigation.

In this work, we investigate how universal MLIPs can be used to obtain material-specific, ab initio-accurate models for particularly demanding systems in which their conventional role as weight initialization for fine-tuning may be insufficient or may require a large amount of ab initio fine-tuning data. Rather than treating them primarily as final production potentials, we use them as efficient configuration space generators for the construction of material-specific MLIPs. In this workflow, a universal model is first used to generate long molecular dynamics trajectories at a low cost. The trajectory is then subsampled, and only the selected structures are recalculated with DFT. The final material-specific potential is trained or fine-tuned exclusively on the DFT-labeled configurations. This separation of sampling and labeling is essential: errors in the universal model energies and forces do not directly enter the training labels, while the universal model still provides an inexpensive access to broad and physically meaningful regions of configuration space. The approach therefore exploits the transferability of universal MLIPs without relying on their zero-shot predictions to be quantitatively accurate.

This strategy is related to active-learning, on-the-fly learning, and self-training pipelines, in which configurations are generated with an inexpensive model, selected, relabeled with first-principles calculations, and used to retrain a specialized potential.^52–56^ It differs from them in two respects that are central to this work. First, the generator is a pretrained state-of-the-art universal model rather than a potential that is built up during the loop, so that a broad and physically meaningful region of configuration space is accessible already from the first trajectory. Second, the configurations are selected by equidistant subsampling, that is, without an uncertainty criterion, a collective variable, or any system-specific descriptor, which keeps the protocol identical across chemically unrelated materials and fixes the first-principles budget in advance. Our aim is accordingly not to replace uncertainty-driven selection, which remains a powerful tool, but to establish quantitatively how far the simplest system-independent protocol carries across a broad benchmark and to identify the observables for which it does not suffice.

We systematically assess this strategy across seven chemically distinct systems that span different bonding motifs, phases, and dynamical processes: cesium dihydrogen phosphate (CDP) and its derivative Cs_7_(H_4_PO_4_)­(H_2_PO_4_)_8_ (CPP),^57–59^ l-pyroglutamate-ammonium (L-Pyro),^9,49,60,61^ solvated phenol (PhOH), aqueous potassium hydroxide (KOH),^62^ crystalline Li_13_Si_4_,^10,63,64^ and MoS_2_ ^34,65,66^ containing sulfur vacancies. These systems probe nonequilibrium properties and rare and reactive events: proton transport, low-barrier hydrogen bonds, liquid-phase solvation, concentrated electrolytes, intermetallic bonding, and defect dynamics in a two-dimensional material. For each system, a target observable defines the accuracy criterion against which the material-specific models are judged. For CDP and CPP, the activation energies of proton diffusion have to reproduce the correct ordering *E*_a_(CDP) < *E*_a_(CPP). For aqueous KOH, the hydroxide diffusion coefficient has to exceed that of water by a factor of about three. For Li_13_Si_4_, the lithium diffusion coefficient has to agree with the DFT value to within ±0.01 Å^2^/ps. For solvated PhOH, the solvation structure has to be consistent with the DFT reference in the positions and heights of the first two peaks and of the first minimum of the radial distribution function. For l-pyroglutamate-ammonium, the double-well shape of the proton-transfer profile has to be recovered with a barrier within ±0.01 eV of the DFT value. For MoS_2_, the sulfur-vacancy migration profile has to show the correct overall shape and a barrier height within ±0.1 eV of the DFT reference. A more detailed description of the systems and the corresponding target observables is provided in previous work which established this set of systems to evaluate the one-shot fine-tuning performance of various MLIP architectures.^35^ We first evaluate the zero-shot performance of nine universal MLIPs, ranging from earlier foundation models to state-of-the-art models such as mace-omat-0,^67^ grace-2l-oam,^30^ pet-oam-xl,^68^ and pet-mad-s-v1.5.0.^69,70^ We then compare material-specific models obtained by fine-tuning on data sets generated either from AIMD trajectories or from DFT-recalculated universal-model trajectories.

Together, these results establish practical guidelines for constructing material-specific MLIPs from a limited amount of DFT data. Universal MLIPs are most reliable when used to accelerate the data set generation process: they provide inexpensive access to long and diverse trajectories, while DFT remains responsible for the final reference labels. The resulting material-specific models enable nanosecond-scale, first-principles-quality atomistic simulations within a practical computational workflow while retaining validation against the physical observables of interest. Furthermore, the workflow proposed here can be applied with minimal user input and does not require system-specific procedures.

## State-of-the-Art Universal Models

Universal machine-learning interatomic potentials have rapidly improved in scope, accuracy, and chemical coverage.^31,68^ The first broadly used generation of foundation MLIPs, such as mace-mp-0 (small) and sevennet-0, was largely trained on subsampled materials project trajectory data.^71–73^ More recent universal models extend this training base to substantially larger and more diverse data sets, including structures from the materials project,^71–73^ alexandria,^74,75^ openmaterials2024,^75^ and openmolecules2025.^76^ Together with larger and more advanced model architectures, this has led to a clear improvement in benchmark accuracy.^31^ At the same time, these models have become more expensive at inference, and the central practical question remains whether their improved zero-shot performance is sufficient for target-observable-driven atomistic simulations.

We first examine this question using sulfur-vacancy dynamics in 2D MoS_2_ as a deliberately demanding test case (Figure 1), before broadening the analysis to six additional chemically diverse systems. The sulfur-vacancy jump probes a high-energy, nonequilibrium part of the potential energy surface with a DFT barrier of about 0.8 eV and is therefore sensitive to errors that may not be visible from equilibrium structures or small distortions alone. This process is representative of regimes in which MLIPs are particularly useful, because long simulations are required, while also being among the most challenging cases for zero-shot performance: defect migration, ion diffusion, bond formation and breaking, and solvation in disordered phases.

**Figure 1:**
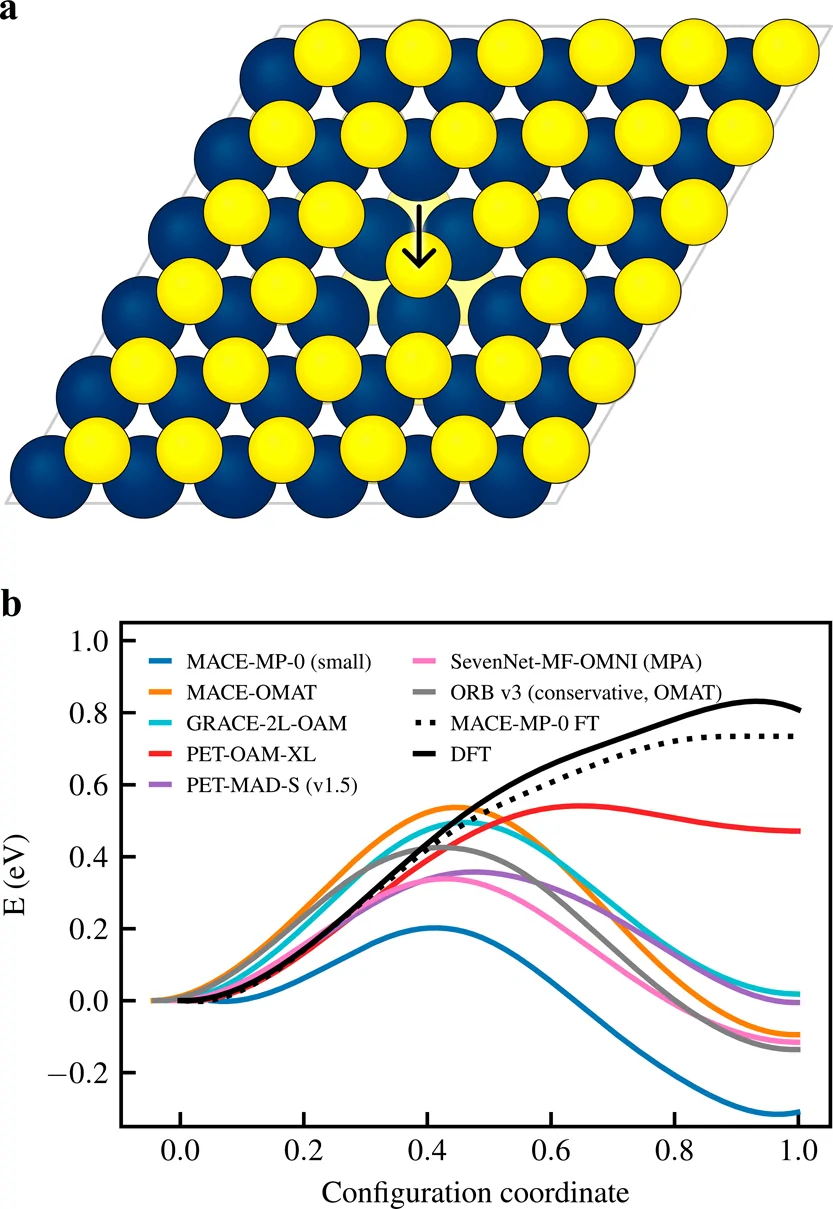
Sulfur-vacancy jump in MoS_2_ and the corresponding energy profile. **a** Sulfur-vacancy jump in a 2D MoS_2_ system. The yellow spheres indicate sulfur atoms, and the blue spheres molybdenum atoms. The black arrow shows the sulfur-vacancy jump investigated in this work, and the gray line indicates the size of the supercell. **b** Potential energy curves for a sulfur jump into a neighboring line of sulfur-vacancies, obtained via nudge elastic band calculations with the different universal models (colored solid lines), one fine-tuned mace-mp-0 foundation model (2000 equidistantly sampled AIMD frames) (black dotted line), and the DFT reference profile^34^ (black solid line).

The universal models predict the potential energy profile of this process with varying accuracy when compared to DFT. Older foundation models can fail qualitatively, while newer and larger models substantially reduce the error but still do not recover the DFT reference profile with the accuracy required for a reliable interpretation. Even the best-performing zero-shot universal model, pet-oam-xl, underestimates the sulfur-vacancy jump barrier by approximately 0.3 eV. Thus, although the newest universal MLIPs are clearly more applicable than earlier models, the improved performance does not yet guarantee ab initio level accuracy for reactive or high-barrier material-specific observables.

In contrast, material-specific adaptation strongly improves the description of the same process. A mace-mp-0 universal model fine-tuned on DFT-labeled MoS_2_ configurations, obtained from subsampled AIMD trajectories, reproduces the DFT energy profile substantially more accurately than the corresponding zero-shot universal model.

To test whether this conclusion is specific to MoS_2_ or general across chemical space, we evaluated nine universal models on seven chemically distinct systems and compared their ability to reproduce material-specific observables. The tested models span both earlier and state-of-the-art universal MLIPs: mace-mp-0 (small),^27^ grace-1l-oam,^30^ sevennet-0,^77^ mattersim-v1.0.0-5M,^28^ orb-v2,^78^ mace-omat-0 (medium),^67^ grace-2l-oam,^30^ pet-oam-xl,^68^ and pet-mad-s-v1.5.0.^69,70^ The full observable-level comparison is provided in Supplementary Notes 1–9. The corresponding force and energy errors are summarized in Supplementary Notes 10 and 11, and a detailed discussion of the accuracy of the investigated universal models is given in Supplementary Note 12.

Across the seven systems, the direct zero-shot use of universal models remains unreliable when the target quantity depends on rare or reactive events. Some models reproduce selected observables for selected materials, but no universal model consistently reaches the desired accuracy across all of the tested cases. This comparison establishes the central motivation of this work: universal MLIPs are not always reliable as final production potentials, but they may provide an efficient starting point for constructing accurate material-specific models.

## Using Universal Models to Sample the Configuration Space

The main bottleneck in constructing material-specific MLIPs is the generation of representative first-principles reference data. A straightforward conventional strategy, besides active learning, is to run AIMD simulations, subsample the resulting trajectories, and train or fine-tune a model on the selected configurations. This approach is robust, but it becomes costly when long trajectories, large simulation cells, different concentrations, or broad temperature ranges are required. Moreover, AIMD spends first-principles effort on every simulation step, although only a small fraction of the generated structures are ultimately used for training.

We therefore use universal MLIPs as low-cost training-structure generators. In this workflow, a state-of-the-art universal model is first used to produce long molecular dynamics trajectories of the target material. The trajectory is then subsampled, and only the selected configurations are recalculated with DFT. The final material-specific model is trained or fine-tuned exclusively on the DFT-labeled structures. In this way, the universal model is not required to reproduce the target observable quantitatively in a zero-shot mode. Its role is instead to generate physically meaningful structures that cover relevant regions of phase space at a substantially lower cost than AIMD.

This separation of sampling and labeling is essential. Errors in the universal model energies and forces do not directly enter the training labels because all selected configurations are recalculated with DFT. The final accuracy is therefore controlled by the diversity and relevance of the sampled structures, together with the quality of the DFT reference labels. Consequently, a universal model can be valuable for data set generation even when it is not accurate enough for direct zero-shot predictions. The level of theory used for labeling can also be changed straightforwardly, for example, by recalculating the selected configurations with hybrid-functional DFT energies and forces.

Throughout this work, the configurations are selected by equidistant subsampling rather than by an uncertainty criterion, and this is a deliberate design choice. Uncertainty-driven selection requires an ensemble of models to be trained, which multiplies the training cost by the committee size, introduces a selection threshold that has to be tuned for every system, and is not uniformly available across the MLIP frameworks compared here; the committee spread is moreover only a proxy for the actual deviation from the first-principles reference.^55,79^ While uncertainty-driven selection remains a powerful tool, equidistant sampling has three properties that are decisive for the present purpose. It is system-independent, requiring neither a collective variable nor a descriptor nor a reaction coordinate, so that the identical protocol applies to a crystalline proton conductor, a liquid electrolyte, and a defective two-dimensional material. It fixes the first-principles budget a priori since the number of DFT single-point calculations is chosen in advance instead of emerging from a convergence criterion. And because the selection does not depend on any intermediate model, all single-point calculations are mutually independent and can be executed as a single embarrassingly parallel batch, whereas active-learning loops are inherently sequential and alternate between training, sampling, selection, and labeling.

Modern universal models generate such trajectories at a small fraction of the cost of AIMD, and the associated computational budget can be stated explicitly. For the CDP system (512 atoms), mace-omat-0 (medium)^67^ produces 500,000 MD steps in 40 h on a single nvidia A100 (40 GB, 400 W) GPU. Subsampling 2000 configurations from this trajectory and recalculating them with CP2K at the PBE/DZVP-MOLOPT/GTH level costs 0.4 core-hours per configuration. Because these single-point calculations are independent, they parallelize trivially: distributed over four 32-core nodes, they are completed in 7 h of wall time, whereas generating the same 500,000 steps by AIMD would occupy that allocation for several weeks. Fine-tuning mace-mp-0 (small) on the resulting data set for 200 epochs adds 2 h on one A100, so that a material-specific, ab initio-labeled model is available after roughly 2 days. A production trajectory of 1,000,000 ab initio-quality MD steps can then be generated with the fine-tuned model in 28 h on the same GPU hardware, resulting in a complete workflow from data set generation to production trajectory in 3 days.

We first compare mace-mp-0 models fine-tuned on conventional AIMD data with models fine-tuned on DFT-recalculated structures generated by universal model trajectories (Figure 2). For the 2000-point data sets, trajectories generated with mace-omat-0 (medium) (Figure 2b), grace-2l-oam (Figure 2c), and pet-oam-xl (Figure 2d) lead to force errors comparable to the AIMD-trained reference models (Figure 2a) for CDP, CPP, KOH, Li_13_Si_4_, and L-Pyro. The corresponding energy errors, shown in Supporting Information Note 13, are generally higher for the universal-model trajectory data sets than for the AIMD data sets. However, they mostly remain below 0.005 eV/atom, compared to values mostly below 0.003 eV/atom for the AIMD-based models. The behavior is less favorable for MoS_2_ and PhOH. For these systems, models trained on structures sampled from universal-MLIP trajectories show force errors that are approximately two to three times larger than those of models trained on subsampled AIMD data. The difference in energy error is also more pronounced.

**Figure 2:**
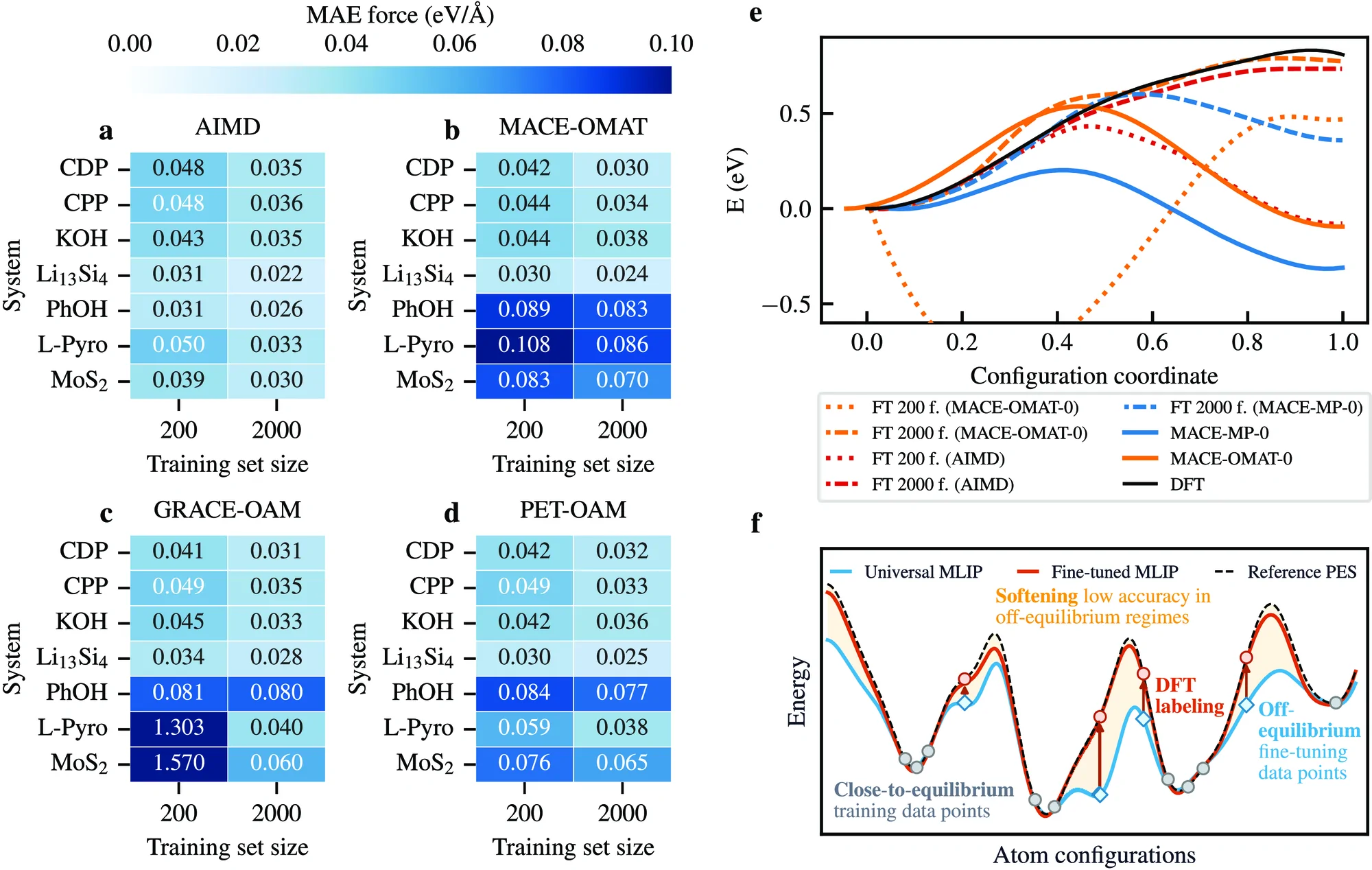
Fine-tuning on universal model sampled structures. Force mean-absolute error in eV/Å of mace-mp-0 models fine-tuned on training sets (200 and 2000 data points) obtained by subsampled AIMD trajectories (**a**); DFT recalculated universal model structures subsampled from 3 ns MD trajectories calculated with mace-omat-0 (medium) (**b**), grace-2l-oam (**c**), and pet-oam-xl (**d**) for different systems (CDP, CPP, KOH, Li_13_Si_4_, L-Pyro, MoS_2_, and PhOH). The corresponding energy errors, normalized per atom, are shown in Supplementary Note 13. **e** Potential energy curves for a sulfur jump into a neighboring line of sulfur-vacancies, obtained via nudged elastic band calculations with the fine-tuned models on 200 data points (dotted lines) and 2000 data points (dashed lines) from AIMD (red lines), mace-mp-0 (blue lines), and mace-omat-0 (orange lines); mace-mp-0 foundation model (solid blue line) and DFT (solid black line). The corresponding NEB profiles including uncertainty estimates are shown in Supplementary Note 29. **f** Schematic illustration of the systematic softening of a one-dimensional potential energy surface by a universal MLIP. The DFT potential energy surface is shown as a dashed black line, while the softened potential energy surface predicted by the universal model is shown in blue. The orange-shaded region highlights the systematic softening error of the universal model in the high-energy regime. Gray circles indicate configurations represented in the universal-model training data. Blue diamonds indicate high-energy configurations sampled during molecular dynamics with the universal model; after DFT relabeling, these configurations are shown as red circles. The potential energy surface obtained after fine-tuning the MLIP on the relabeled configurations is shown in red.

The training set size has a strong effect on the final model quality. As expected, increasing the data set from 200 to 2000 DFT-labeled configurations improves the models, with the strongest influence observed for the energy accuracy. With only 200 data points, several models remain insufficiently accurate, and no stable fine-tuned mace-mp-0 model could be obtained for L-Pyro and MoS_2_ when DFT-labeled structures were generated by grace-2l-oam.

The MoS_2_ sulfur-vacancy jump provides the most stringent test of whether the resulting models reproduce the target potential energy surface because this process has a high barrier of 0.8 eV, which renders transition-state configurations statistically rare and therefore highly sensitive to errors in the learned energy landscape. Among the fine-tuned mace-mp-0 models shown in Figure 2e, only the models fine-tuned on 2000 data points from either subsampled AIMD trajectories or DFT-recalculated mace-omat-0 trajectory structures reproduce the DFT energy profile with reasonable accuracy. In contrast, a model fine-tuned on 2000 DFT-recalculated structures generated by the older and less accurate mace-mp-0 foundation model does not recover the DFT curve. The 200 data point models also fail to reproduce the correct profiles. Thus, both the quality of the structure-generating model and the size of the DFT-labeled data set are decisive. Repeating these fine-tunings with five independent seeds for the train/validation split and the mini-batch selection shows that the models that recover the DFT barrier do so with an ensemble spread that is small compared with the differences to other models, whereas the seed dependence is more pronounced for the models that do not (Supporting Information Note 29). The same holds for the iteratively fine-tuned models of Figure 3a (Supporting Information Note 30).

**Figure 3:**
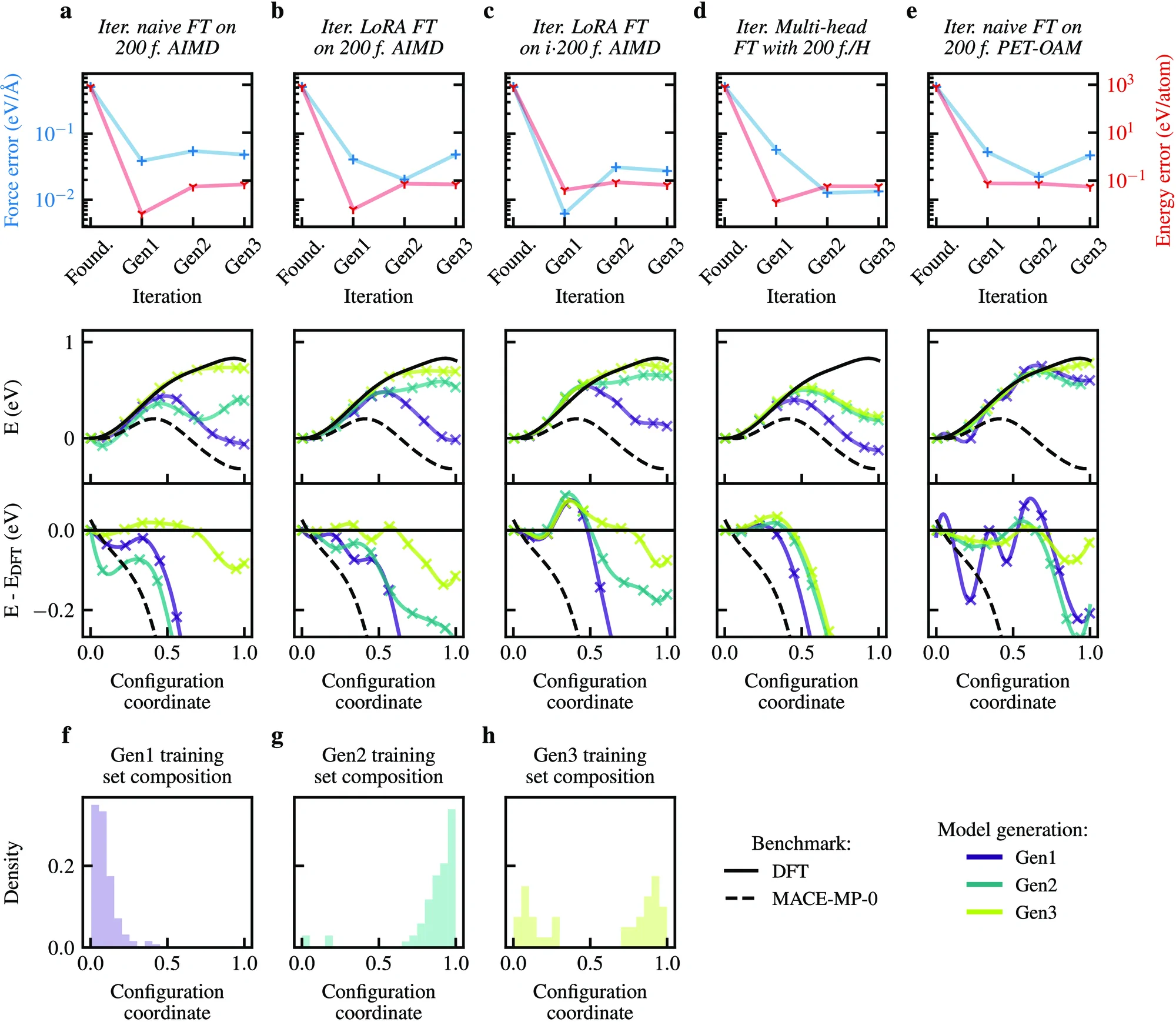
Iterative self-training. Evaluation of three generations of iteratively fine-tuned mace-mp-0 models for MoS_2_. From top to bottom, the rows show force and energy errors, predicted energy profiles of the sulfur-vacancy jump in MoS_2_, deviations from the DFT reference profile and histograms of the training data composition. Different iterative fine-tuning strategies are compared: **a** naive fine-tuning initialized with 200 AIMD data points and a constant data set size of 200 points per model; **b** LoRA fine-tuning initialized with 200 AIMD data points and a constant data set size of 200 points per model; **c** LoRA fine-tuning initialized with 200 AIMD data points and a data set size increasing linearly by 200 points per generation; **d** multihead replay fine-tuning initialized with 200 AIMD data points, using 200 data points per generation and one head per generation; **e** naive fine-tuning initialized with 200 DFT-labeled structures obtained by subsampling a pet-oam-xl universal-model trajectory, using a constant data set size of 200 points per model. NEB profiles including uncertainty estimates for the iteratively trained models of **a** are shown in Supplementary Note 30. The DFT reference profile is shown as a solid black line, and the corresponding universal model energy profile is indicated by a black dashed black line. The data set composition for the model shown in **a** for each generation is indicated by the colored bars in the lower panels **f**–**h** for generations 1–3, respectively, with respect to the configuration coordinate.

The corresponding sulfur-vacancy energy profiles for models fine-tuned on DFT-recalculated grace-2l-oam and pet-oam-xl trajectories are shown in Supporting Information Note 14. As for the mace-omat-0-based data sets, the models trained on 2000 data points recover the correct qualitative and quantitative behavior. This confirms that the target observable is considerably more sensitive than the average force error. A model can reach a reasonable global error while still failing for a specific high barrier process if the relevant configurations are not sufficiently represented in the training data. This is particularly evident for the sulfur-vacancy jump in MoS_2_: the force and energy errors are ensemble-averaged quantities over all atoms and configurations, whereas the NEB profile is governed by a localized displacement of a single sulfur atom along a high-energy migration pathway. Consequently, small average errors over the full validation set do not necessarily imply an accurate description of the local potential energy surface that controls this jump process.^79^

We further evaluated whether models trained on universal model-generated structures reproduce the material-specific observables of the remaining systems. The results for mace-mp-0 models fine-tuned on mace-omat-0-generated data sets are summarized in Supporting Information Note 15, and the corresponding results for pet-oam-xl-generated data sets are shown in Supporting Information Note 16. With the exception of the hydroxide ion mobility, the relevant physical properties are reproduced accurately by fine-tuned models. This demonstrates that universal-model-generated data sets can be sufficient for a broad range of material-specific observables, although difficult transport or reaction processes can still require additional care. For comparison, the observables obtained from mace-mp-0 models fine-tuned on AIMD data sets are given in Supporting Information Note 17.

We then used the DFT-labeled mace-omat-0 data set to fine-tune additional universal models instead of mace-mp-0, namely, sevennet-0, mattersim-v1.0.0-5M, grace-1l-oam, and orb-v2. The results are shown in Supporting Information Notes 18–22. As discussed in our previous work, fine-tuning these models with a reasonably large data set results in material-specific models with comparable performance largely independent of architecture.^35^ For MoS_2_, L-Pyro, and PhOH, however, the fine-tuned models show elevated errors that are approximately two to three times larger than those of the AIMD-based fine-tuned models, similar to the fine-tuned mace-mp-0 models for these materials in Figure 2b.

The ability of these different fine-tuned universal models to predict the sulfur-vacancy jump energy curve in MoS_2_ is shown in Supporting Information Note 20. All models based on the DFT-labeled 2000-point mace-omat-0 data set reproduce the energy profile, except for the orb-v2 model. The performance of the sevennet-0 and mattersim-v1.0.0-5M models for the material properties of the other systems is shown in Supporting Information Notes 23 and 24. These material-specific models reproduce the observables for most systems but do not predict the hydroxide ion diffusivity with the desired first-principles accuracy.

Finally, we considered training models from scratch instead of fine-tuning the universal models. For this comparison, mace models of similar size to the mace-mp-0 foundation model were trained from scratch on the same data sets. The force and energy errors are shown in Supporting Information Notes 23 and 24. For the 2000-point data sets, accurate trained-from-scratch models are obtained for CDP, CPP, KOH, and Li_13_Si_4_ when using structures generated by pet-oam-xl, mace-omat-0 (medium), or grace-2l-oam. For L-Pyro, similarly accurate models are obtained with grace-2l-oam and mace-omat-0. The resulting errors are generally comparable to those of models trained from scratch on subsampled AIMD data and are often smaller than those of the corresponding fine-tuned mace-mp-0 models shown in Figure 2a–d. For the MoS_2_ sulfur-vacancy jump, the energy profiles obtained with trained-from-scratch models show particularly robust behavior, as shown in Supporting Information Note 25.

While using universal MLIPs to sample configurational space, we are exploiting the recently discussed systematic softening of the potential energy surface.^41^ In this context, softening refers to the tendency of universal models to underestimate energy barriers in high-energy regions of the potential energy surface, as illustrated in Figure 2f. This behavior likely originates from the composition of the training data, which is typically dominated by close-to-equilibrium structures. Although this limits the direct use of universal MLIPs for quantitative barrier predictions, it can be advantageous for data set generation: softened barriers allow the model to visit off-equilibrium configurations more frequently during molecular dynamics. After these configurations are relabeled with first-principles calculations, they provide targeted reference data for training a material-specific MLIP with improved accuracy in the high-energy regions that are underrepresented in the equilibrium AIMD data.

However, this strategy is not guaranteed to be successful in a single step. If the generated trajectory does not sample the relevant transition region sufficiently, or if the relabeled data set still lacks configurations controlling the target observable, then the resulting model may retain the same qualitative failure. These model failures therefore identify cases where a single-pass data set-generation strategy is insufficient and naturally motivate an iterative refinement procedure.

## Iterative Training

The previous results show that DFT-recalculated universal-model trajectories provide an efficient route to accurate material-specific MLIPs but also that a single data set-generation step is not always sufficient. This limitation becomes particularly important when the target observable depends on a narrow, rare, or high-energy region of the configuration space. For such cases, we investigate an iterative training workflow in which the current material-specific model is used to generate structures for the next training iteration.

The procedure is conceptually simple. In the first generation, a model is trained from scratch or fine-tuned using an initial DFT-labeled data set obtained either from a subsampled AIMD trajectory or from a DFT-recalculated subsampled universal-MLIP trajectory. This model was then used to run a molecular dynamics simulation. The resulting trajectory is equidistantly subsampled, and the selected configurations are recalculated with DFT. These newly labeled configurations are then used to fine-tune the current model and obtain the next model generation, either as a replacement for the previous data set or in combination with data from earlier generations. The loop is repeated until the target observable is reproduced with the desired accuracy. In analogy to modern large-language-model workflows, this strategy can be viewed as iterative self-training or synthetic data generation;^80^ however, in the present case, every newly generated structure is relabeled with first-principles calculations before it enters the training set. This approach is particularly promising because MLIPs trained primarily on equilibrium structures show the tendency to underestimate the potential energies of transition states,^41^ making such states more likely to appear in training sets sampled from MLIP-based MD simulations than from AIMD simulations.

We demonstrate this strategy for the most challenging observable considered in this work: the sulfur-vacancy jump in MoS_2_. The initial model is obtained by fine-tuning mace-mp-0 on only 200 configurations subsampled from an AIMD trajectory (Figure 3a). This first-generation model is then used to perform an MLIP molecular dynamics simulation of 600,000 steps. From this trajectory, 200 configurations are equidistantly sampled and recalculated with DFT to form the training set for the next-generation. Repeating this procedure yields a third-generation model that correctly reproduces the DFT sulfur-vacancy energy profile while requiring only 600 first-principles single-point calculations in total and two additional MLIP molecular dynamics simulations.

The composition of the successive training sets makes the underlying mechanism directly visible. Figure 3f–h shows the distribution of the sampled structures along the configuration coordinate of the sulfur-vacancy jump for the three generations. The initial data set, subsampled from the AIMD trajectory, is confined to configuration coordinates between 0 and approximately 0.25 (Figure 3f): the DFT barrier keeps the system close to the equilibrium state, and the high-energy region is not reached within an affordable trajectory length and training set size. The first-generation model fine-tuned on these structures consequently underestimates the energy of the final configuration severely, and the molecular dynamics simulation performed with it spends most of its time beyond the barrier; the resulting data set is dominated by configuration coordinates between 0.5 and 1.0 (Figure 3g). This is softening-assisted sampling in its most explicit form: the error of the generating model is what opens access to the transition and product regions that the AIMD data lack. Because these structures are relabeled with DFT, the second-generation model raises the energy of the final configuration substantially, although it still underestimates it. Its residual softening is now moderate, so the trajectory generated with it visits both sulfur-vacancy sites and the corresponding data set covers configuration coordinates around 0.1 and around 0.9 in a balanced way (Figure 3h). The third-generation model trained on this data set reproduces the DFT profile. The progression from Figure 3f to Figure 3h therefore shows quantitatively how the softening is exploited generation by generation and how it is simultaneously reduced by the first-principles data it makes available.

This result is notable because models trained on data sets containing 600 AIMD configurations or 600 mace-mp-0-generated configurations fail to reproduce the DFT profile, as shown in Supporting Information Note 26. Even fine-tuning on 2000 DFT-labeled configurations sampled with the older mace-mp-0 foundation model does not recover the correct curve (Figure 3e).

We further investigated whether alternative fine-tuning strategies improved the iterative workflow. When the same iterative procedure is performed with LoRA fine-tuning instead of naive fine-tuning (Figure 3b), the third-generation model reaches a performance comparable to that of the naively fine-tuned model. However, the second-generation LoRA model already predicts the sulfur-vacancy energy profile substantially more accurately than the corresponding naively fine-tuned model. This indicates that LoRA fine-tuning can improve data efficiency in the early stages of iterative training, where the available DFT-labeled data sets are still small.

We then tested whether retaining data from previous generations further improved the LoRA-based workflow. In this variant, the data set size increases linearly by 200 structures per generation. For example, the second-generation model is fine-tuned not only on the 200 newly generated configurations but also on a combined data set containing the initial AIMD-based structures and the newly DFT-labeled structures generated from the first-generation model, resulting in 400 data points in total. This strategy leads to a substantially improved second-generation prediction of the sulfur-vacancy energy profile (Figure 3c). The third-generation model, however, performs comparably to the iterative models trained with a constant training data set size of 200 points per generation.

In contrast, multihead replay fine-tuning does not provide a clear additional benefit for this system. In this strategy, each newly generated data set is assigned to a separate head, with one head per model generation (Figure 3d). Although the second-generation model improves relative to the initial model, no substantial additional improvement is observed in the third-generation model. This suggests that for the present MoS_2_ case, separating the generations into different heads is less effective than directly adapting the shared model parameters to the newly sampled configuration space.

Finally, we tested whether the iterative workflow requires an AIMD-based initial data set. Starting from 200 DFT-labeled structures obtained by subsampling a pet-oam-xl universal-model trajectory already yields a well-performing first-generation model (Figure 3e). Additional iterative fine-tuning further improves the model initially, demonstrating that the workflow can also be initialized from universal-model-generated structures.

However, during further iterations, the model accuracy decreases, as shown in Supporting Information Note 27. This behavior can be rationalized from the data set composition of the respective training sets, shown in Supporting Information Note 28, and indicates that iterative self-training must still be monitored with respect to the diversity and relevance of the generated configurations. The workflow automates the generation and labeling of the training data, not the scientific validation, since the final model must always be tested against the physical observable of interest, and the convergence of the iterative refinement must be monitored critically.

Together, these results show that iterative training provides a practical fallback when one-shot fine-tuning or training on AIMD- or universal-model-generated data sets does not yet reach the required target accuracy. The strategy is particularly useful when the relevant configurations are not sufficiently represented in the initial data set.

## Training Guidelines

This work provides an extensive, target-observable-driven benchmark of state-of-the-art universal MLIPs used as configuration-space generators, a comparative analysis of nine foundation models across seven reactive systems, and a set of practical guidelines derived from them. We systematically compared zero-shot universal models, naive fine-tuning, training from scratch, and iterative fine-tuning across seven chemically distinct systems and several families of modern MLIPs. The study deliberately focuses on broadly accessible strategies based on naive fine-tuning and on data set construction through equidistant sampling of molecular dynamics trajectories. This allows the resulting guidelines to be applied across different MLIP frameworks without relying on specialized active learning infrastructure. Two results go beyond this benchmarking role. The first is the systematic comparison of iterative self-training against one-shot fine-tuning at matched first-principles budget, which shows that repeatedly regenerating the configuration space is markedly more data-efficient than enlarging a single data set. The second is the identification of the softening of universal potential energy surfaces as a usable sampling asset rather than merely a source of error and the demonstration that an iterative loop exploits this bias progressively while the DFT labels regularize the unphysical regions it also explores.

The first conclusion is that state-of-the-art universal MLIPs should generally not be treated as final production potentials for target observable-driven simulations. Although the newest models are substantially more accurate than earlier generations, they still fail for several observables connected to reactive events, ion mobility, defect motion, or high-energy regions of the potential energy surface. The sulfur-vacancy jump in MoS_2_ is the clearest example: even the best zero-shot universal model underestimates the barrier by approximately 0.3 eV. For mechanistic interpretation, this level of error is too large because relevant observables depend exponentially on the energy barrier according to Boltzmann statistics, and ab initio-level accuracy cannot be assumed for universal models in such cases.

The second conclusion is that universal models are, nevertheless, highly valuable. Their most robust role is not necessarily to replace DFT directly but to generate diverse and physically meaningful configurations at low computational cost. After DFT recalculation of selected frames, these structures form accurate material-specific training data sets. Using state-of-the-art universal MLIPs as structure generators enables the exploration of nanosecond-scale trajectories before deciding which configurations should receive expensive first-principles labeling. This shifts the data-generation bottleneck from continuous AIMD to a set of independent DFT single-point calculations that can be parallelized efficiently.

For the systems investigated here, 2000 DFT-recalculated configurations selected from trajectories generated by modern universal MLIPs are often sufficient to obtain accurate material-specific models. When state-of-the-art universal models are used to generate the structures, approximately two-thirds of the investigated cases lead to fine-tuned mace-mp-0 models with the desired accuracy. Counting over the 21 system/generator combinations investigated (seven systems, each sampled with three different state-of-the-art universal MLIPs), 14 of 21 fine-tuned mace-mp-0 models reach the accuracy criterion defined in the Methods section on the test set. Using the same data sets for training from scratch, 13 of 21 models satisfy the identical criterion. Here, training from scratch performs on par with or better than naive fine-tuning when the data set contains 2000 configurations. This identifies an important advantage of universal models: even when their zero-shot predictions are not sufficiently accurate, they can strongly accelerate the generation of useful material-specific training data. These assignments rest on single training runs, as detailed in the Methods section. For the two central MoS_2_ results we therefore repeated the fine-tunings with five different seeds for the train/validation split and the mini-batch selection, which indicates that the models reproducing the DFT barrier are only weakly seed-dependent, so that the reported classification is unlikely to be an artifact of a single realization (Supporting Information Notes 29 and 30).

The quality of the structure-generating model is critical. Data sets generated with older or less accurate universal MLIPs can lead to substantially worse material-specific models, even when all selected structures are relabeled with DFT. This is because the DFT labels correct the energies and forces of the sampled configurations but cannot compensate for missing regions in the sampling of the configuration space. If the generating model does not visit the relevant structures, then the final material-specific model cannot learn them. This effect is especially important for rare events and transition pathways, where average force and energy errors alone may not reveal failure. As universal models continue to improve, the fraction of systems for which this workflow yields accurate material-specific models is expected to increase.

The results suggest the following practical workflow.

1 . When no AIMD trajectory is available, start by generating a trajectory with a state-of-the-art universal MLIP, such as pet-oam-xl, mace-omat-0 (medium) or grace-2l-oam. Subsample approximately 2000 configurations from this trajectory and recalculate these structures with parallelized DFT single-point calculations. The resulting data set was used to train a material-specific model from scratch or to fine-tune a suitable foundation model. Based on the present results, mace and sevennet are particularly useful choices for training from scratch, while mace-mp-0, sevennet-0, and mattersim-v1.0.0-5M provide strong fine-tuning baselines.
2 . The resulting model should be validated on the physical observable of interest, not only on global force and energy errors. For many systems, a one-shot data set generated from a modern universal-model trajectory is sufficient. However, observables such as the MoS_2_ sulfur-vacancy jump show that good average errors do not always imply correct target behavior. Target-observable validation is therefore essential, especially for high-barrier processes, transport properties, and reaction mechanisms.
3 . If the one-shot model fails, then apply iterative self-training. Use the current material-specific model to generate additional trajectories, subsample the configurations it visits, recalculate them with DFT, and continue training or fine-tuning. This strategy can be more efficient than simply increasing the size of the initial data set because it exploits a potential softening of the MLIP,^41^ which can automatically lead to an overrepresentation of particularly relevant transition states in the training set. For MoS_2_, this approach recovers the correct sulfur-vacancy energy profile using only 600 DFT single-point calculations in total, whereas several noniterative strategies with comparable or larger data sets fail.

Overall, universal MLIPs provide the greatest benefit when used to accelerate the data set generation process. They allow long and diverse trajectories to be generated cheaply, while DFT remains responsible for final reference labels. Material-specific MLIPs trained on these data sets can then reach the accuracy required for ab initio-quality simulations at a fraction of the cost of direct AIMD. The resulting workflow enables nanosecond-scale, first-principles-quality atomistic trajectories within only a few days while retaining a clear validation path through target observables and iterative refinement when necessary.

## Methods

### AIMD Simulations

Reference DFT AIMD simulations were performed on CPUs using CP2K (version 2025.1)^81–87^ with the BLYP (KOH, L-Pyro, PhOH)^88,89^ and PBE (CDP, CPP, MoS_2_, Li_13_Si_4_)^90^ exchange–correlation functional, GTH pseudopotentials,^91–93^ DZVP-MOLOPT basis set,^94^ and Nosé–Hoover chain thermostats^95–97^ applying a time step of 0.5 fs. Training data consisted of 10, 200, and 2000 configurations per system equidistantly sampled from AIMD trajectories at relevant temperatures: 300 K (PhOH, L-Pyro), 333 K (KOH), 500 K (Li_13_Si_4_), 510, 540, 585, 620, and 660 K (CDP, CPP), and 1000 K (MoS_2_) with a stride of 10 and 100.

### Training and Fine-Tuning MLIPs

All fine-tuning and training calculations were performed using the respective MLIP framework implementations: mace-torch (version 0.3.14),^20,21^ grace tensorpotential (version 0.5.1),^24,30^ sevennet (version 0.11.2),^47,77^ mattersim (version 1.1.2),^28^ and orb (version 0.3.2)^29,78^ through their official releases. For fine-tuning we employed the smaller and more efficient models of the respective MLIP frameworks: mace-mp-0 (small),^27^ grace-1l-oam,^30^ sevennet-0,^77^ mattersim-v1.0.0-5M (MatterSim-Large),^28^ and orb-v2.^78^ For training, we used the same model sizes as the mentioned smaller foundation models, to be able to compare model performance between fine-tuning and training. Fine-tuning and training were performed using NVIDIA A100 GPUs.

To assess the sensitivity of the principal results to stochastic training effects, the sulfur-vacancy energy profiles shown in Figure 2e and Figure 3a were each repeated using five independent random seeds (1–5). The resulting ensemble means and standard deviations are reported in Supporting Information Notes 29 and 30. For the remainder of the benchmark, each reported model was obtained from a single training run with a fixed seed (1). Given the scale of the study, comprising several hundred individual training and fine-tuning runs across different frameworks, seven systems, and three universal potential models, extending the complete benchmark to multiple seeds would have increased an already substantial computational cost by the size of the ensemble. Moreover, in fine-tuning the network parameters are inherited from a pretrained foundation model rather than randomly initialized, so different seeds do not correspond to independent starting points in parameter space. Residual seed dependence instead arises through the train/validation split, the ordering of mini-batches, and any newly initialized parameters, as in LoRA or multihead replay fine-tuning. Consequently, all reported errors and pass/fail assignments outside the five-seed analysis correspond to the reference realization obtained with seed 1.

For production and the training set generated from DFT-recalculated foundation model structures, we used more sophisticated and expensive models: mace-omat-0 (medium),^27^ grace-2l-oam,^30^ sevennet-omni (mpa modal),^98^ pet-oam-xl,^68,99^ and pet-mad-s-v1.5.0.^69,70,99^

All errors reported in this work were computed on reference data excluded from the training and validation sets. This test data set was obtained by recalculating, with first-principles methods, MLIP trajectories generated using the fine-tuned foundational mace-mp-0 models (2000 data points, subsampled AIMD trajectories, stride 100). Throughout this work, a model is considered to have reached the desired accuracy if it satisfies both MAE(F)<0.05⁡eV/Å and MAE­(*E*) < 0.006 eV/atom with respect to the DFT reference, evaluated on the test data set described above. These thresholds are not chosen ad hoc: they correspond to the accuracy typically reached by fine-tuned universal MLIPs as reported in the literature,^26,27^ and thus define a model that is as accurate as fine-tuning is generally expected to be. The same two thresholds are applied uniformly to every system, every configuration-generating universal model, and both training strategies; no system-specific tolerances are used.

### MLIP MD and NEB Calculations

Molecular dynamics simulations were performed with ase (version 3.25.0)^100^ using Nosé–Hoover chain thermostats^95–97^ set up with amaceing_toolkit (version 0.7.0)^35,101^ for 3 ns at 300 K (PhOH, L-Pyro), 333 K (KOH), 500 K (Li_13_Si_4_), 510, 540, 585, 620, and 660 K (CDP, CPP), and 1000 K (MoS_2_) with a time step of 0.5 fs. Nudged elastic band calculations were performed with ase (version 3.25.0).^100^ MD and NEB calculations were performed using nvidia A100 GPUs.

## Supplementary Material



## Data Availability

The data set containing training sets and the models are available at doi.org/10.5281/zenodo.20792778.

## Notes

Absolute force and energy errors for universal and fine-tuned MLIP models across all systems; mean-squared displacements, molecular dynamics analysis for physical observable prediction analyses: diffusion coefficients, radial-distribution functions, proton-transfer free energy profiles, and potential energy curve analyses; evaluation of fourth generation of iterative models; uncertainty estimations for main results (PDF)

## Notes

The used third-party codes cp2k, ase, mace, mattersim, grace, sevennet, upet and orb are available at cp2k.org, ase-lib.org, github.com/acesuit/mace, github.com/microsoft/mattersim, github.com/ICAMS/grace-tensorpotential, github.com/MDIL-SNU/SevenNet, github.com/lab-cosmo/upet, and github.com/orbital-materials/orb-models, respectively. The input-script creator used in this study is available at github.com/jhaens/amaceing_toolkit.
