# Supplementary material for: Universal Interatomic Potentials as Configuration-Space Generators for One-Shot and Iterative Fine-Tuning of Ab Initio-Accurate Material-Specific Models
Source: J Phys Chem Lett. 2026 Aug 26;17(36):10349–61. doi: 10.1021/acs.jpclett.6c02067 (PMC13573618; doi:10.1021/acs.jpclett.6c02067)
Supplement: Supplementary file 1 [file jz6c02067_si_001.pdf]

Supplementary Information for "Universal Interatomic Potentials as Configuration-Space Generators for One-Shot and Iterative Fine-Tuning of Ab Initio-Accurate Material-Specific Models"

Jonas Hänseroth,[1,*] Aaron Flötotto,[1] and Christian Dreßler[1]

[1] *Theoretical Solid State Physics, Institute of Physics, Technische Universität Ilmenau, 98693 Ilmenau, Germany*

(Dated: August 6, 2026)

For more information on abbreviations, please refer to the main text, where all abbreviations are defined in detail. Abbreviations not introduced in the main text are defined here.

Supplementary Note 1: MACE-MP-0 (small) universal model - prediction of physical properties 3

Supplementary Note 2: MatterSim-v1.0.0-5M universal model - prediction of physical properties 4

Supplementary Note 3: SevenNet-0 universal model - prediction of physical properties 5

Supplementary Note 4: GRACE-1L-OAM universal model - prediction of physical properties 6

Supplementary Note 5: ORB-v2 universal model - prediction of physical properties 7

Supplementary Note 6: MACE-OMAT-0 (medium) universal model - prediction of physical properties 8

Supplementary Note 7: GRACE-2L-OAM universal model - prediction of physical properties 9

Supplementary Note 8: PET-OAM-XL universal model - prediction of physical properties 10

Supplementary Note 9: PET-MAD-S-v1.5.0 universal model - prediction of physical properties 11

Supplementary Note 10: Universal model performance - Force errors 12

Supplementary Note 11: Universal model performance - Energy errors 13

Supplementary Note 12: Discussion of universal model performances 14

Supplementary Note 13: Fine-tuned MACE model performance - Energy errors - Dataset generating universal model vs. systems vs. dataset size (universal model dataset) 15

Supplementary Note 14: Fine-tuned model (universal model dataset) - prediction of potential energy profile for sulfur-vacancy jump in MoS_2 16

Supplementary Note 15: MACE-MP-0 fine-tuned universal model (MACE-OMAT dataset) - prediction of physical properties 17

Supplementary Note 16: MACE-MP-0 fine-tuned universal model (PET-OAM dataset) - prediction of physical properties 18

Supplementary Note 17: MACE-MP-0 fine-tuned universal model (AIMD dataset) - prediction of physical properties 19

Supplementary Note 18: Fine-tuned model performance - Force errors - MLIPs vs. systems vs. dataset size (MACE-OMAT universal model dataset) **20**

Supplementary Note 19: Fine-tuned model performance - Energy errors - MLIPs vs. systems vs. dataset size (MACE-OMAT universal model dataset) **21**

Supplementary Note 20: Fine-tuned model (MACE-OMAT-0 model dataset) - prediction of potential energy profile for sulfur-vacancy jump in MoS_2 **22**

Supplementary Note 21: SevenNet-0 fine-tuned universal model (MACE-OMAT dataset) - prediction of physical properties **23**

Supplementary Note 22: MatterSim-Large fine-tuned universal model (MACE-OMAT dataset) - prediction of physical properties **24**

Supplementary Note 23: Trained MACE model performance - Force errors - Dataset generating universal model vs. systems vs. dataset size (universal model dataset) **25**

Supplementary Note 24: Trained MACE model performance - Energy errors - Dataset generating universal model vs. systems vs. dataset size (universal model dataset) **26**

Supplementary Note 25: Trained-from-scratch model (universal model dataset) - prediction of potential energy profile for sulfur-vacancy jump in MoS_2 **27**

Supplementary Note 26: Comparing iterative training against single time fine-tuning - prediction of potential energy profile for sulfur-vacancy jump in MoS_2 **28**

Supplementary Note 27: Iterative training additional model generations **29**

Supplementary Note 28: Comparing iterative training dataset composition **30**

Supplementary Note 29: Fine-tuned model performance with respect to the configuration space sampling method- NEB energy profiles with uncertainty estimation **31**

Supplementary Note 30: Iterative fine-tuning performance with respect to the configuration space sampling method - NEB energy profiles with uncertainty estimation **32**

References **33**

SUPPLEMENTARY NOTE 1: MACE-MP-0 (SMALL) UNIVERSAL MODEL - PREDICTION OF PHYSICAL PROPERTIES

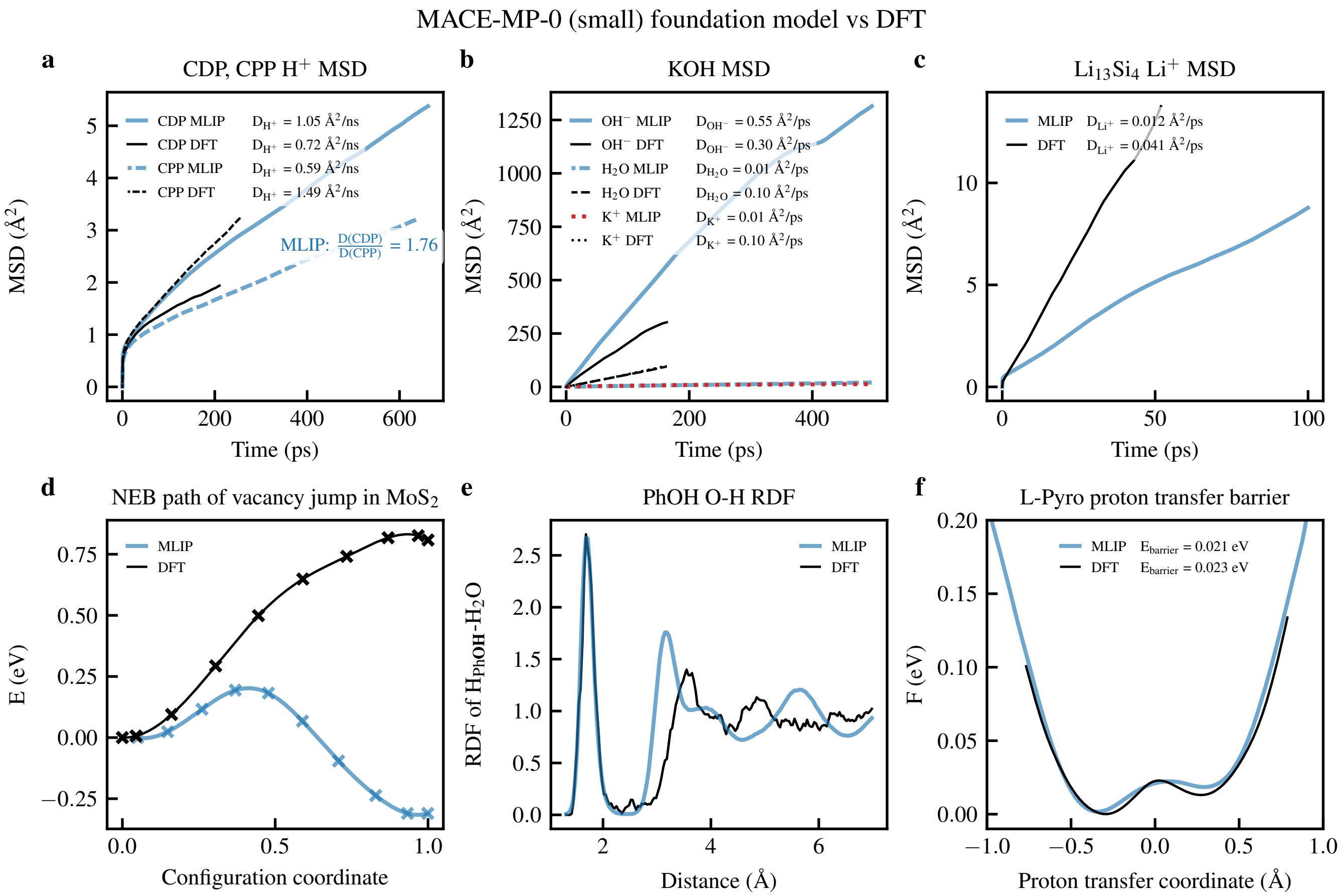

Supplementary Figure 1. **MACE-MP-0 (small) universal model**. Comparison of different physical properties obtained with first-principles methods and the universal model: **a** CsH_2PO_4 (CDP) and $Cs_7(H_4PO_4)(H_2PO_4)_8$ (CPP) - proton diffusion coefficients ratios of D(CDP)/D(CPP), **b** KOH - water, water molecule and hydroxide ion mean-squared displacements and diffusion coefficients, **c** $Li_{13}Si_4$ - lithium ion mean-squared displacements and diffusion coefficients, **d** MoS_2 - potential energy curves for a sulfur jump into a neighboring line of sulfur vacancies, **e** phenol in water - $(H_2O)\cdots O_{\text{Hydroxyl-Group}}$ radial distribution function and **f** L-pyroglutamate-ammonium (L-Pyro) - free energy profiles along the proton transfer coordinate.

SUPPLEMENTARY NOTE 2: MATTERSIM-V1.0.0-5M UNIVERSAL MODEL - PREDICTION OF PHYSICAL PROPERTIES

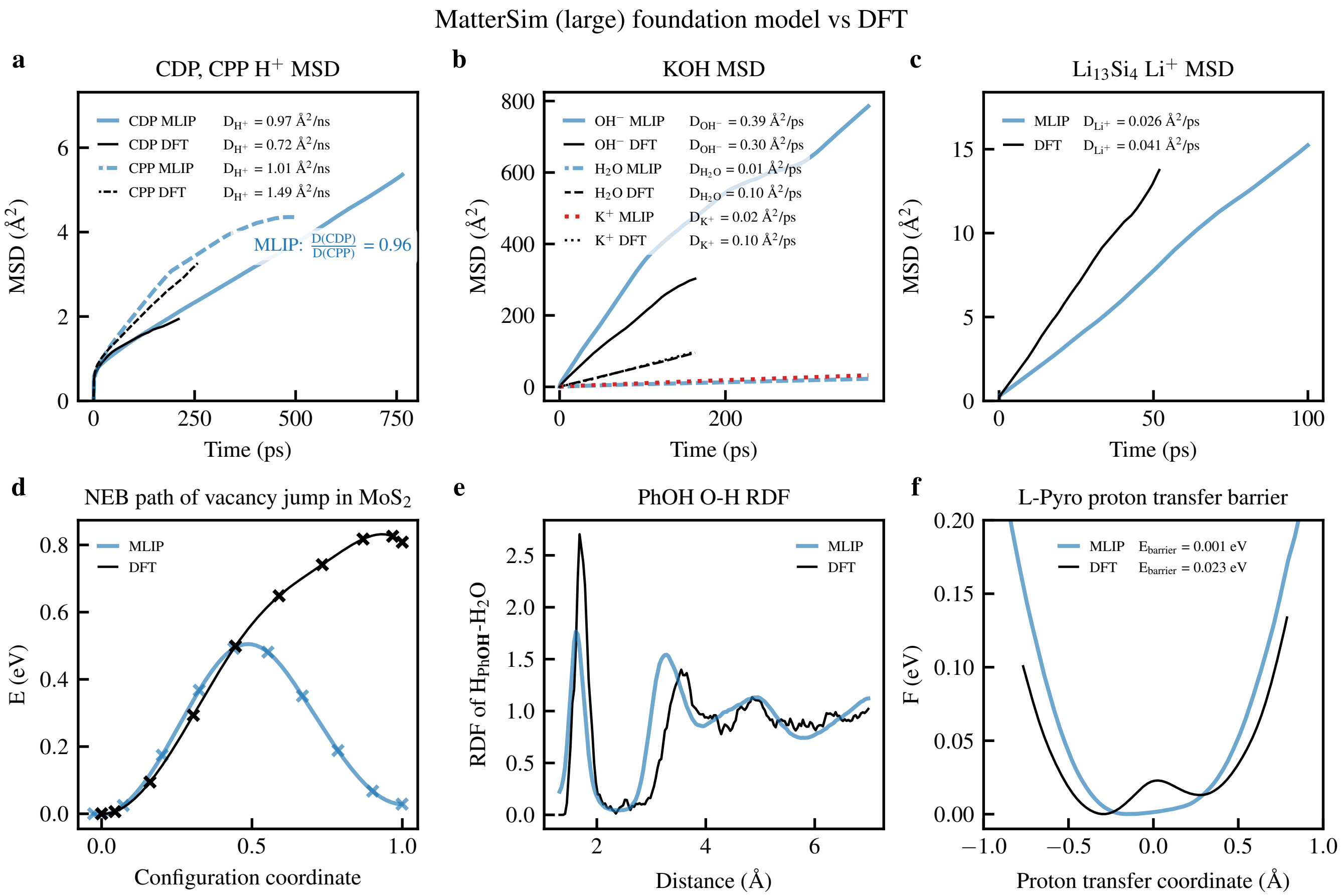

Supplementary Figure 2. **MatterSim-v1.0.0-5M universal model**. Comparison of different physical properties obtained with first-principles methods and the universal model: **a** CsH_2PO_4 (CDP) and $Cs_7(H_4PO_4)(H_2PO_4)_8$ (CPP) - proton diffusion coefficients ratios of D(CDP)/D(CPP), **b** KOH - water, water molecule and hydroxide ion mean-squared displacements and diffusion coefficients, **c** $Li_{13}Si_4$ - lithium ion mean-squared displacements and diffusion coefficients, **d** MoS_2 - potential energy curves for a sulfur jump into a neighboring line of sulfur vacancies, **e** phenol in water - $(H_2O)\cdots O_{\text{Hydroxyl-Group}}$ radial distribution function and **f** L-pyroglutamate-ammonium (L-Pyro) - free energy profiles along the proton transfer coordinate.

SUPPLEMENTARY NOTE 3: SEVENNET-0 UNIVERSAL MODEL - PREDICTION OF PHYSICAL PROPERTIES

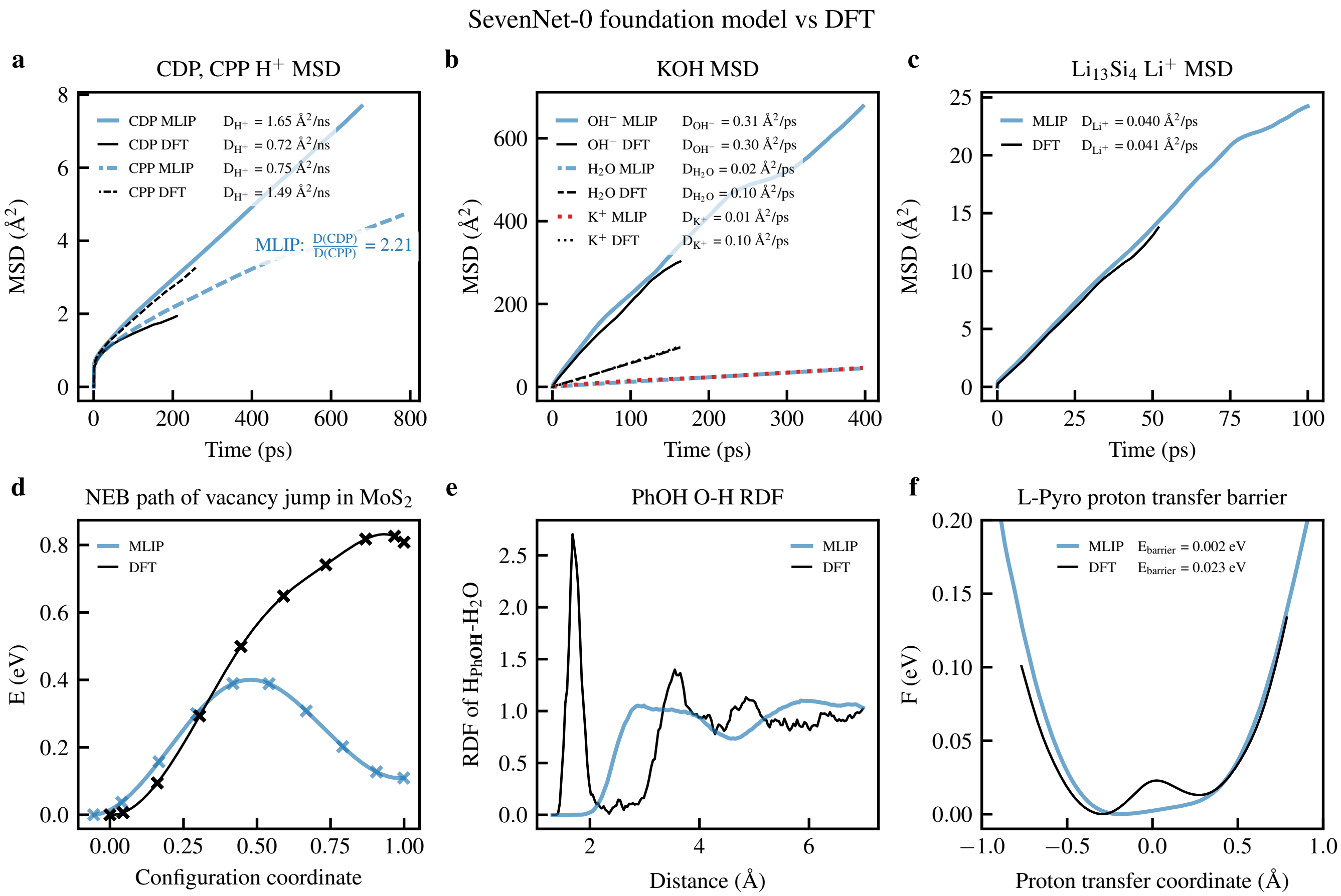

Supplementary Figure 3. **SevenNet-0 (small) universal model**. Comparison of different physical properties obtained with first-principles methods and the universal model: **a** CsH_2PO_4 (CDP) and $Cs_7(H_4PO_4)(H_2PO_4)_8$ (CPP) - proton diffusion coefficients ratios of D(CDP)/D(CPP), **b** KOH - water, water molecule and hydroxide ion mean-squared displacements and diffusion coefficients, **c** $Li_{13}Si_4$ - lithium ion mean-squared displacements and diffusion coefficients, **d** MoS_2 - potential energy curves for a sulfur jump into a neighboring line of sulfur vacancies, **e** phenol in water - $(H_2O)\cdots O_{\text{Hydroxyl-Group}}$ radial distribution function and **f** L-pyroglutamate-ammonium (L-Pyro) - free energy profiles along the proton transfer coordinate.

SUPPLEMENTARY NOTE 4: GRACE-1L-OAM UNIVERSAL MODEL - PREDICTION OF PHYSICAL PROPERTIES

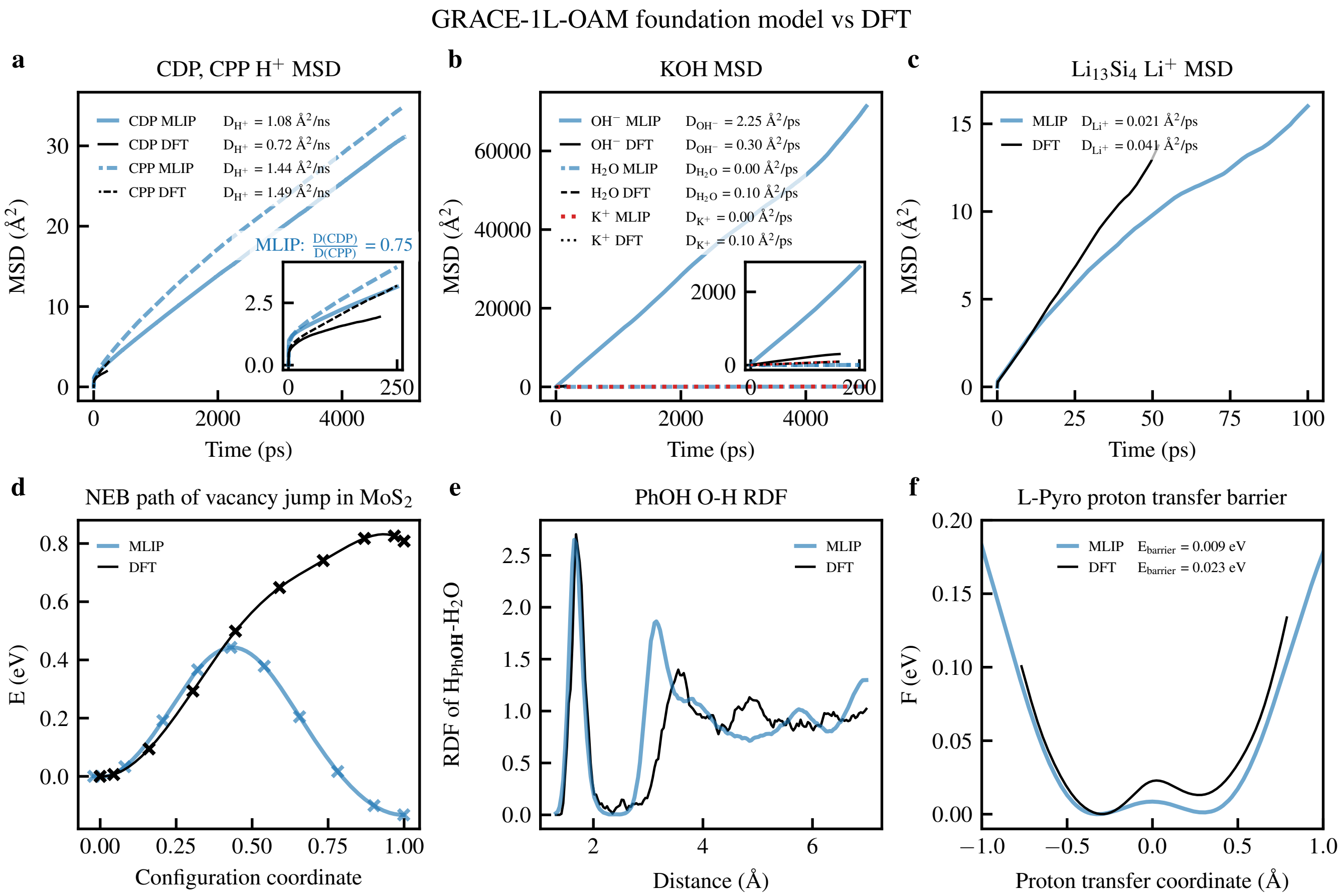

Supplementary Figure 4. **GRACE-1L-OAM universal model**. Comparison of different physical properties obtained with first-principles methods and the universal model: **a** CsH_2PO_4 (CDP) and $Cs_7(H_4PO_4)(H_2PO_4)_8$ (CPP) - proton diffusion coefficients ratios of D(CDP)/D(CPP), **b** KOH - water, water molecule and hydroxide ion mean-squared displacements and diffusion coefficients, **c** $Li_{13}Si_4$ - lithium ion mean-squared displacements and diffusion coefficients, **d** MoS_2 - potential energy curves for a sulfur jump into a neighboring line of sulfur vacancies, **e** phenol in water - $(H_2O)\cdots O_{\text{Hydroxyl-Group}}$ radial distribution function and **f** L-pyroglutamate-ammonium (L-Pyro) - free energy profiles along the proton transfer coordinate.

SUPPLEMENTARY NOTE 5: ORB-V2 UNIVERSAL MODEL - PREDICTION OF PHYSICAL PROPERTIES

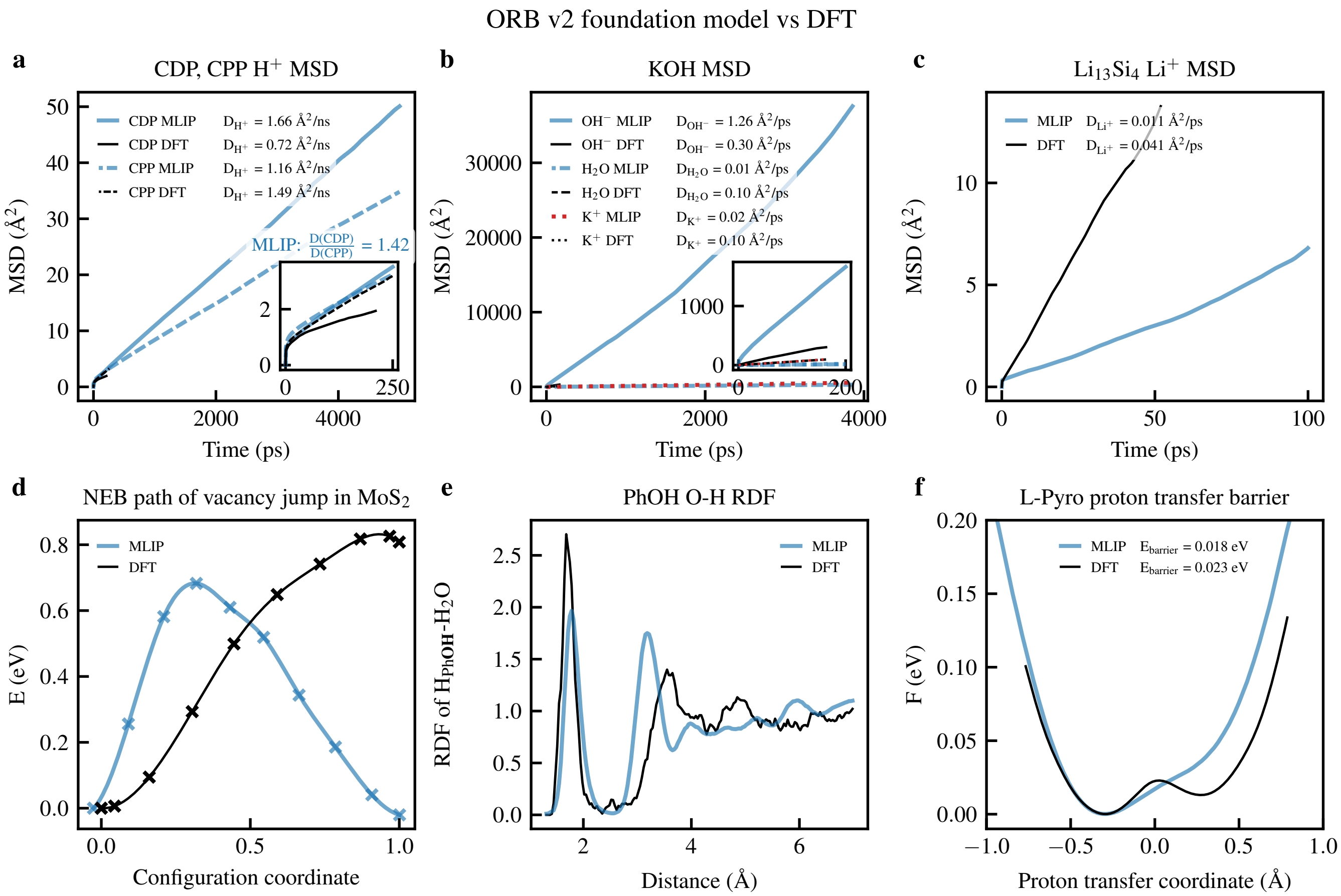

Supplementary Figure 5. **ORB-V2 universal model**. Comparison of different physical properties obtained with first-principles methods and the universal model: **a** CsH_2PO_4 (CDP) and $Cs_7(H_4PO_4)(H_2PO_4)_8$ (CPP) - proton diffusion coefficients ratios of D(CDP)/D(CPP), **b** KOH - water, water molecule and hydroxide ion mean-squared displacements and diffusion coefficients, **c** $Li_{13}Si_4$ - lithium ion mean-squared displacements and diffusion coefficients, **d** MoS_2 - potential energy curves for a sulfur jump into a neighboring line of sulfur vacancies, **e** phenol in water - $(H_2O)\cdots O_{\text{Hydroxyl-Group}}$ radial distribution function and **f** L-pyroglutamate-ammonium (L-Pyro) - free energy profiles along the proton transfer coordinate.

SUPPLEMENTARY NOTE 6: MACE-OMAT-0 (MEDIUM) UNIVERSAL MODEL - PREDICTION OF PHYSICAL PROPERTIES

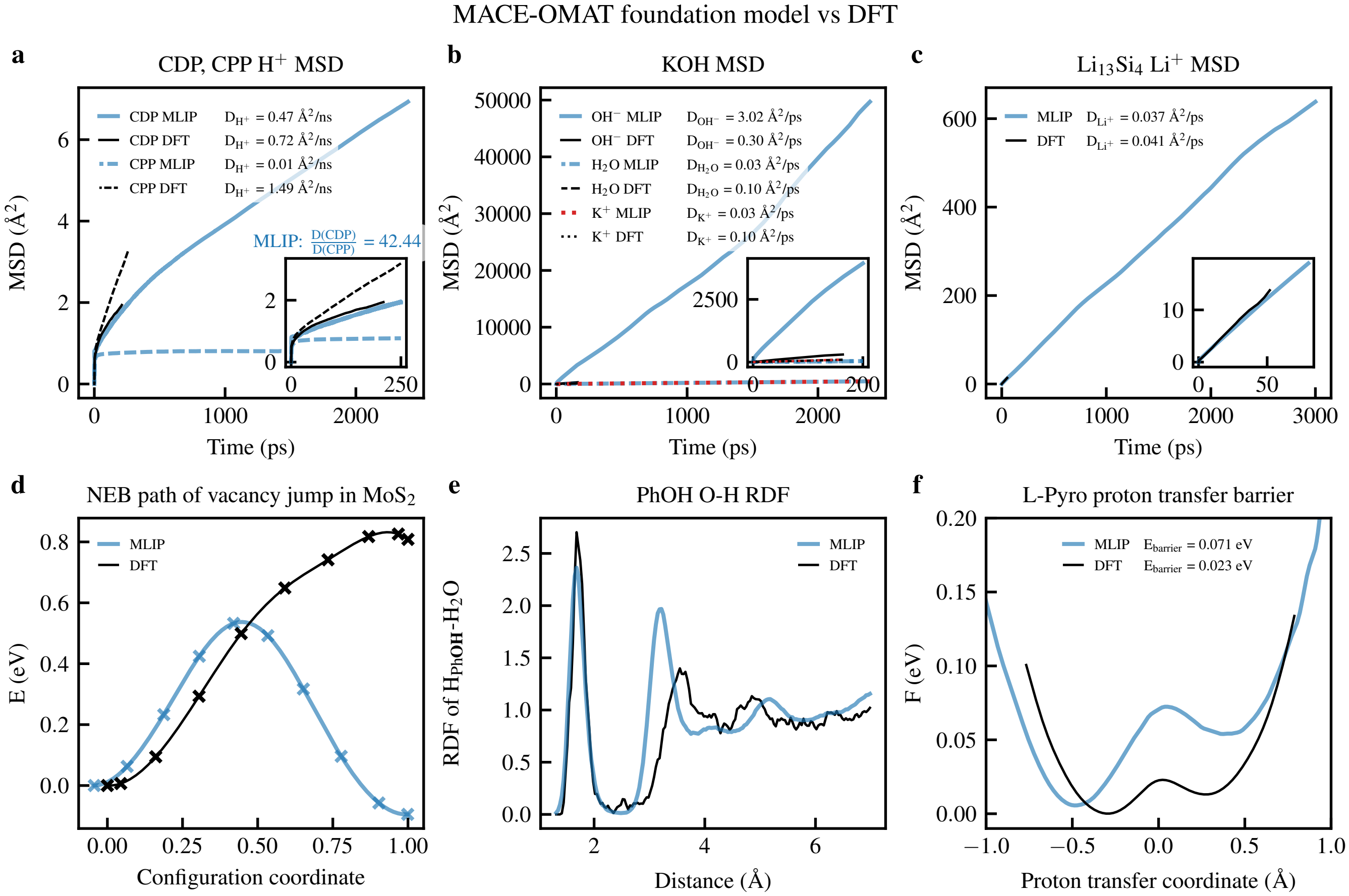

Supplementary Figure 6. **MACE-OMAT-0 (medium) universal model**. Comparison of different physical properties obtained with first-principles methods and the universal model: **a** CsH_2PO_4 (CDP) and $Cs_7(H_4PO_4)(H_2PO_4)_8$ (CPP) - proton diffusion coefficients ratios of D(CDP)/D(CPP), **b** KOH - water, water molecule and hydroxide ion mean-squared displacements and diffusion coefficients, **c** $Li_{13}Si_4$ - lithium ion mean-squared displacements and diffusion coefficients, **d** MoS_2 - potential energy curves for a sulfur jump into a neighboring line of sulfur vacancies, **e** phenol in water - $(H_2O)\cdots O_{\text{Hydroxyl-Group}}$ radial distribution function and **f** L-pyroglutamate-ammonium (L-Pyro) - free energy profiles along the proton transfer coordinate.

SUPPLEMENTARY NOTE 7: GRACE-2L-OAM UNIVERSAL MODEL - PREDICTION OF PHYSICAL PROPERTIES

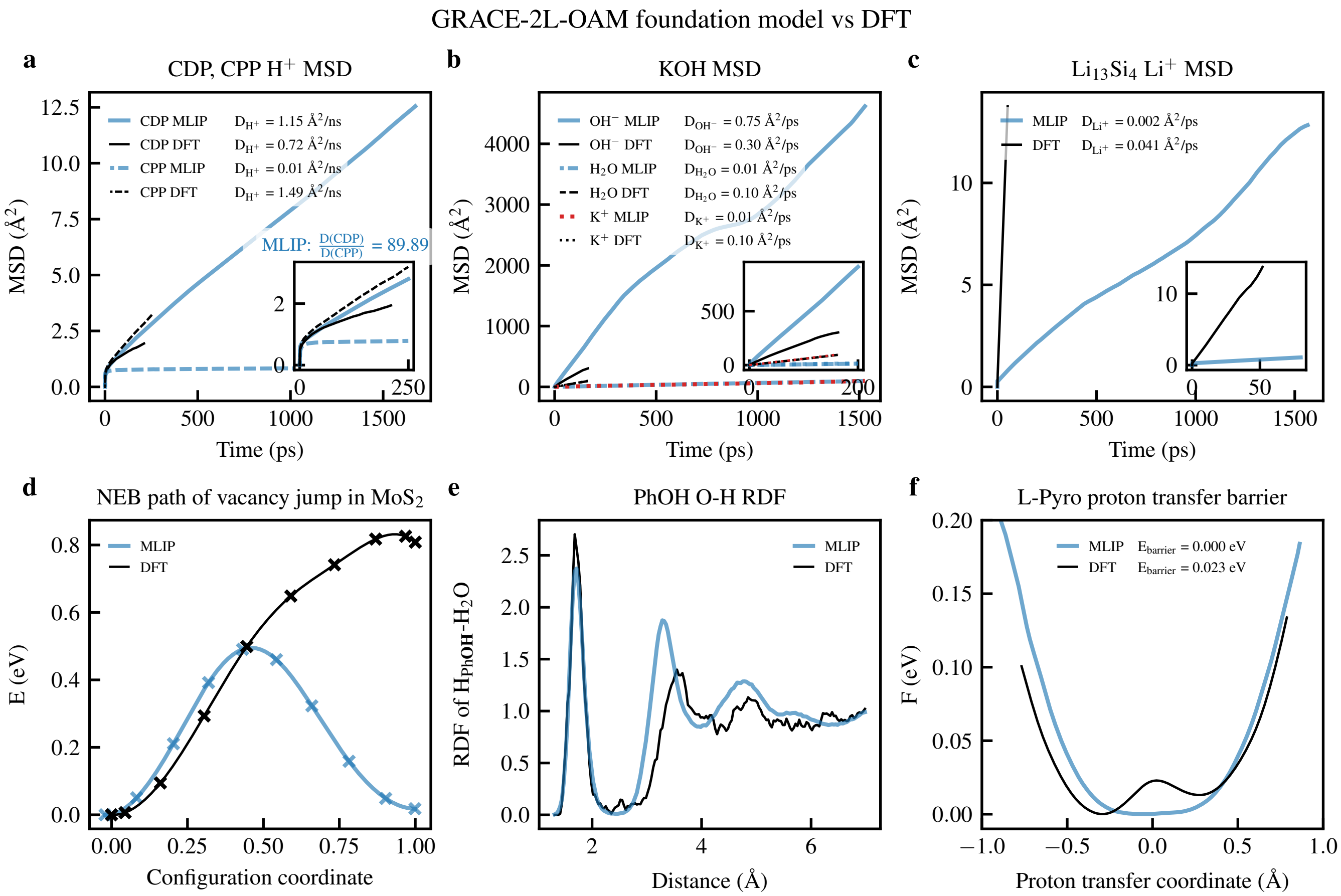

Supplementary Figure 7. **GRACE-2L-OAM universal model**. Comparison of different physical properties obtained with first-principles methods and the universal model: **a** CsH_2PO_4 (CDP) and $Cs_7(H_4PO_4)(H_2PO_4)_8$ (CPP) - proton diffusion coefficients ratios of D(CDP)/D(CPP), **b** KOH - water, water molecule and hydroxide ion mean-squared displacements and diffusion coefficients, **c** $Li_{13}Si_4$ - lithium ion mean-squared displacements and diffusion coefficients, **d** MoS_2 - potential energy curves for a sulfur jump into a neighboring line of sulfur vacancies, **e** phenol in water - $(H_2O)\cdots O_{\text{Hydroxyl-Group}}$ radial distribution function and **f** L-pyroglutamate-ammonium (L-Pyro) - free energy profiles along the proton transfer coordinate.

SUPPLEMENTARY NOTE 8: PET-OAM-XL UNIVERSAL MODEL - PREDICTION OF PHYSICAL PROPERTIES

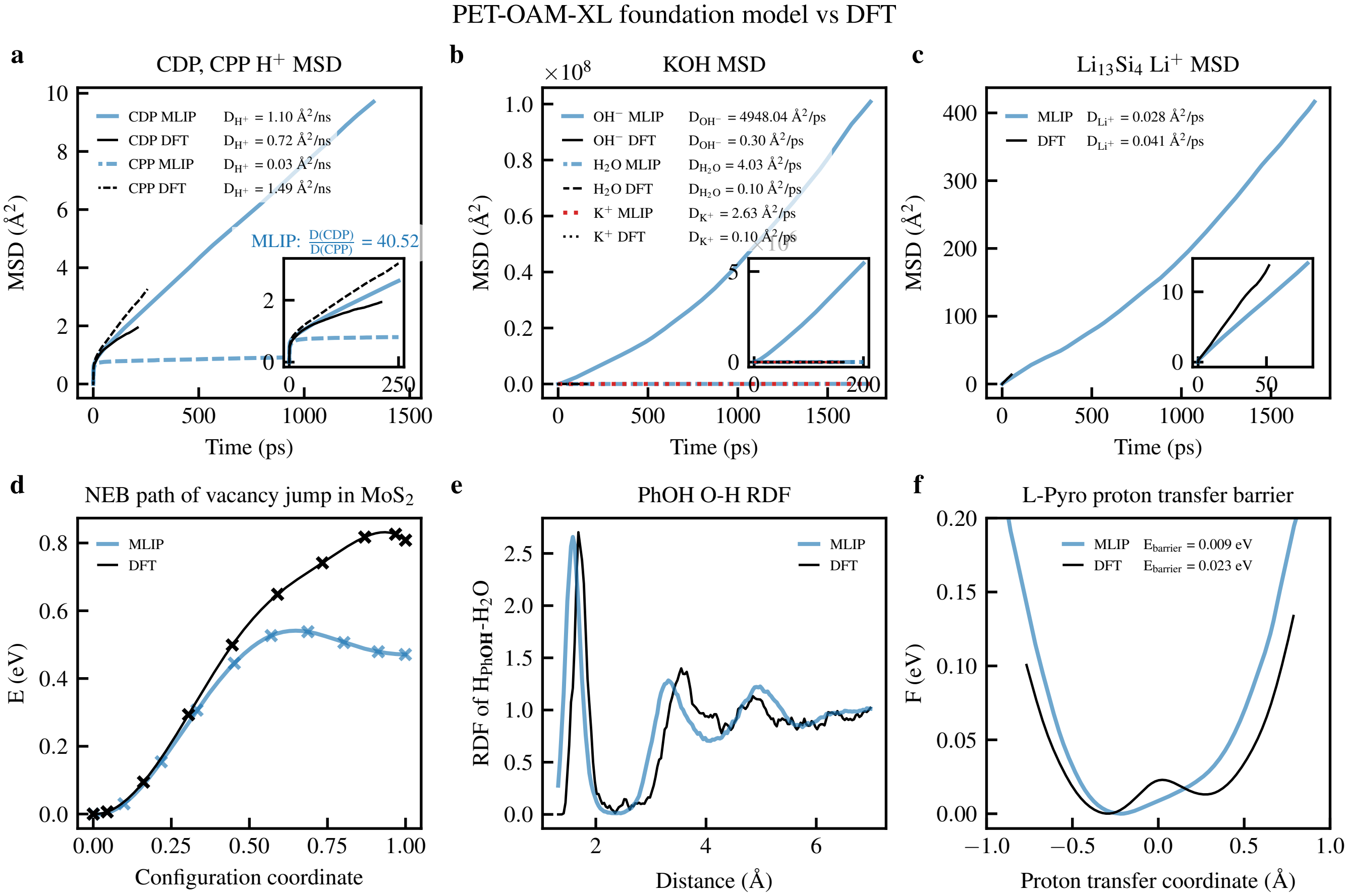

Supplementary Figure 8. **PET-OAM-XL universal model**. Comparison of different physical properties obtained with first-principles methods and the universal model: **a** CsH_2PO_4 (CDP) and $Cs_7(H_4PO_4)(H_2PO_4)_8$ (CPP) - proton diffusion coefficients ratios of D(CDP)/D(CPP), **b** KOH - water, water molecule and hydroxide ion mean-squared displacements and diffusion coefficients, **c** $Li_{13}Si_4$ - lithium ion mean-squared displacements and diffusion coefficients, **d** MoS_2 - potential energy curves for a sulfur jump into a neighboring line of sulfur vacancies, **e** phenol in water - $(H_2O)\cdots O_{\text{Hydroxyl-Group}}$ radial distribution function and **f** L-pyroglutamate-ammonium (L-Pyro) - free energy profiles along the proton transfer coordinate.

SUPPLEMENTARY NOTE 9: PET-MAD-S-V1.5.0 UNIVERSAL MODEL - PREDICTION OF PHYSICAL PROPERTIES

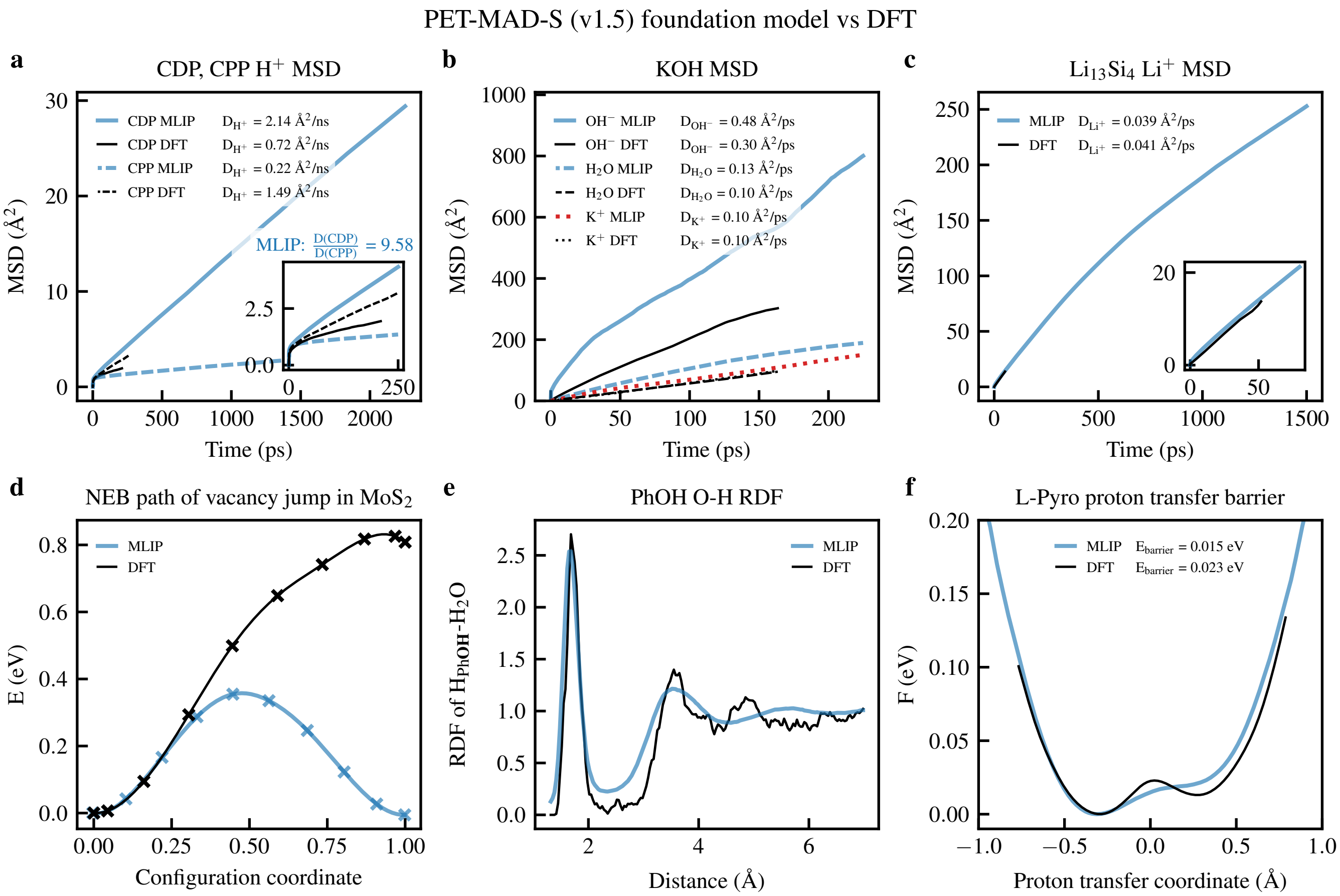

Supplementary Figure 9. **PET-MAD-S-v1.5.0 universal model**. Comparison of different physical properties obtained with first-principles methods and the universal model: **a** CsH_2PO_4 (CDP) and $Cs_7(H_4PO_4)(H_2PO_4)_8$ (CPP) - proton diffusion coefficients ratios of D(CDP)/D(CPP), **b** KOH - water, water molecule and hydroxide ion mean-squared displacements and diffusion coefficients, **c** $Li_{13}Si_4$ - lithium ion mean-squared displacements and diffusion coefficients, **d** MoS_2 - potential energy curves for a sulfur jump into a neighboring line of sulfur vacancies, **e** phenol in water - $(H_2O)\cdots O_{\text{Hydroxyl-Group}}$ radial distribution function and **f** L-pyroglutamate-ammonium (L-Pyro) - free energy profiles along the proton transfer coordinate.

SUPPLEMENTARY NOTE 10: UNIVERSAL MODEL PERFORMANCE - FORCE ERRORS

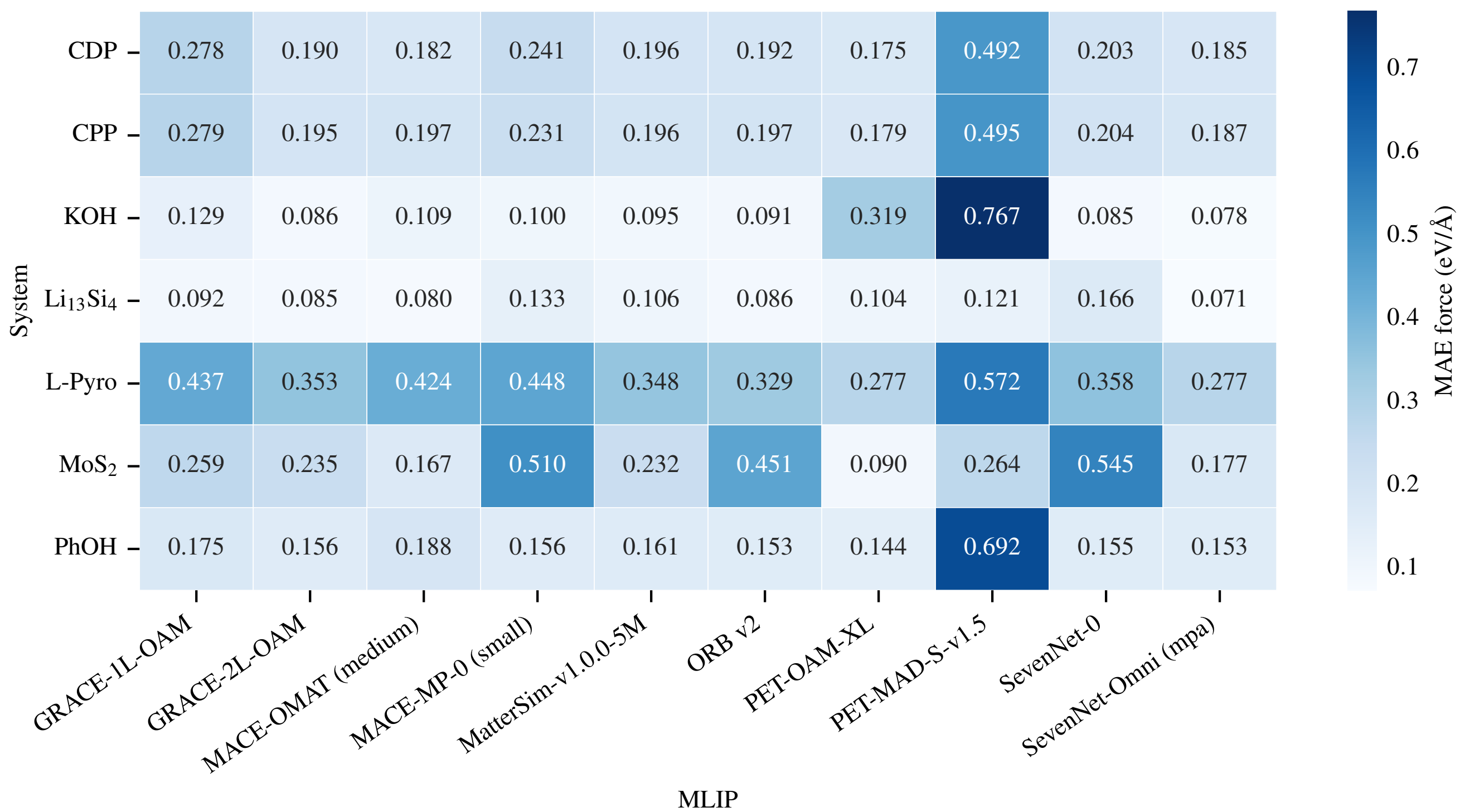

Supplementary Figure 10. **Universal model force errors**. Mean-absolute-error on the predicted forces (in eV/Å) of the different universal MLIPs on the different systems: CsH_2PO_4 (CDP) and $Cs_7(H_4PO_4)(H_2PO_4)_8$ (CPP), aqueous KOH solution, $Li_{13}Si_4$, MoS_2, phenol in water and L-pyroglutamate-ammonium (L-Pyro).

SUPPLEMENTARY NOTE 11: UNIVERSAL MODEL PERFORMANCE - ENERGY ERRORS

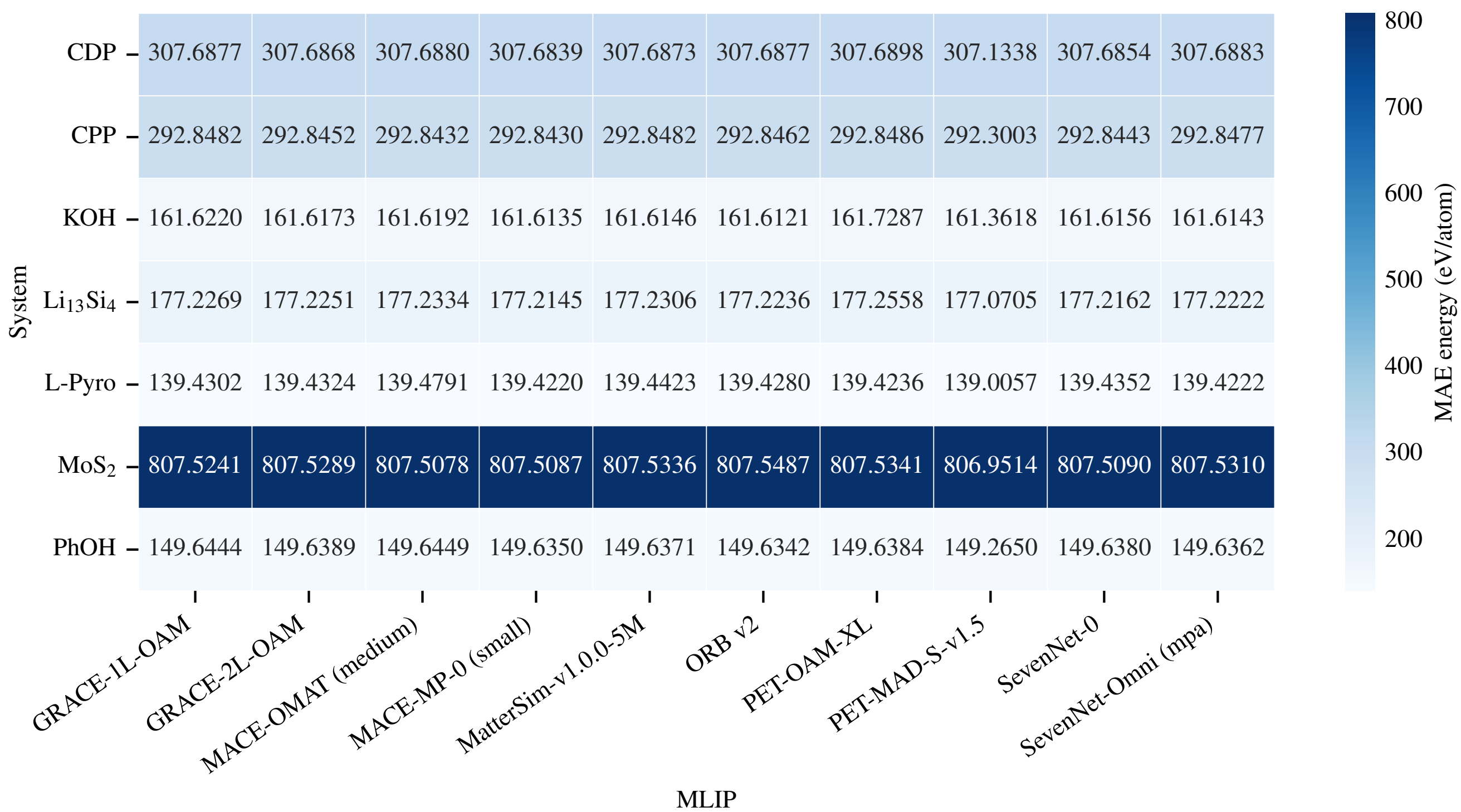

Supplementary Figure 11. **Universal model energy errors**. Mean-absolute-error on the predicted energies of the different universal MLIPs on the different systems: CsH_2PO_4 (CDP) and $Cs_7(H_4PO_4)(H_2PO_4)_8$ (CPP), aqueous KOH solution, $Li_{13}Si_4$, MoS_2, phenol in water and L-pyroglutamate-ammonium (L-Pyro).

SUPPLEMENTARY NOTE 12: DISCUSSION OF UNIVERSAL MODEL PERFORMANCES

A detailed discussion of the performance of the earlier universal models, namely MACE-MP-0 (small) [1], MatterSim-v1.0.0-5M [2], SevenNet-0 [3], GRACE-1L-OAM [4], and ORB-v2 [5], in predicting the physical properties of the seven investigated systems is provided in our previous work [6]. These results are summarized in Supplementary Notes 1-5 and 11-12.

The performance of four newer universal models, namely MACE-OMAT-0 (medium) [7], GRACE-2L-OAM [4], PET-MAD-S-v1.5.0 [8, 9], and PET-OAM-XL [10], in predicting the material-specific properties across the seven systems is shown in Supplementary Notes 6-12.

The force errors reported in Supplementary Note 11 show that these newer universal models perform substantially better than the previously investigated models. For several materials, some of the models achieve force errors below 0.1 eV/Å. Among all investigated models, PET-OAM-XL yields the lowest overall errors, achieving the best performance for five of the seven systems.

PET-OAM-XL also shows the best performance in predicting the energy profile of the sulfur-vacancy jump in MoS_2, although it still does not reach DFT-level accuracy. Furthermore, it is able to reproduce the solvation structure of phenol in water and the lithium-ion diffusion in $Li_{13}Si_4$. These properties are also well described by the MACE-OMAT-0 (medium) universal model.

The PET-MAD-S-v1.5.0 model is able to predict the solvation of the OH group of phenol in water, as well as Li^+ diffusion in $Li_{13}Si_4$ and the mobility of ions and molecules in aqueous potassium hydroxide solution. The GRACE-2L-OAM model reproduces the local solvation environment of the phenol OH group in water and the mobility of K^+ and H_2O in aqueous potassium hydroxide solution, but overestimates the hydroxide-ion diffusion.

Overall, these newer models show improved zero-shot accuracy compared with the previously investigated universal models. This improvement is likely related to their larger and more diverse training datasets, which include data from Alexandria [11, 12], the Materials Project [13–15], Open Materials 2024 [12], and Open Molecules 2025 [16], as well as to the increased number of parameters in these models.

SUPPLEMENTARY NOTE 13: FINE-TUNED MACE MODEL PERFORMANCE - ENERGY ERRORS - DATASET GENERATING UNIVERSAL MODEL VS. SYSTEMS VS. DATASET SIZE (UNIVERSAL MODEL DATASET)

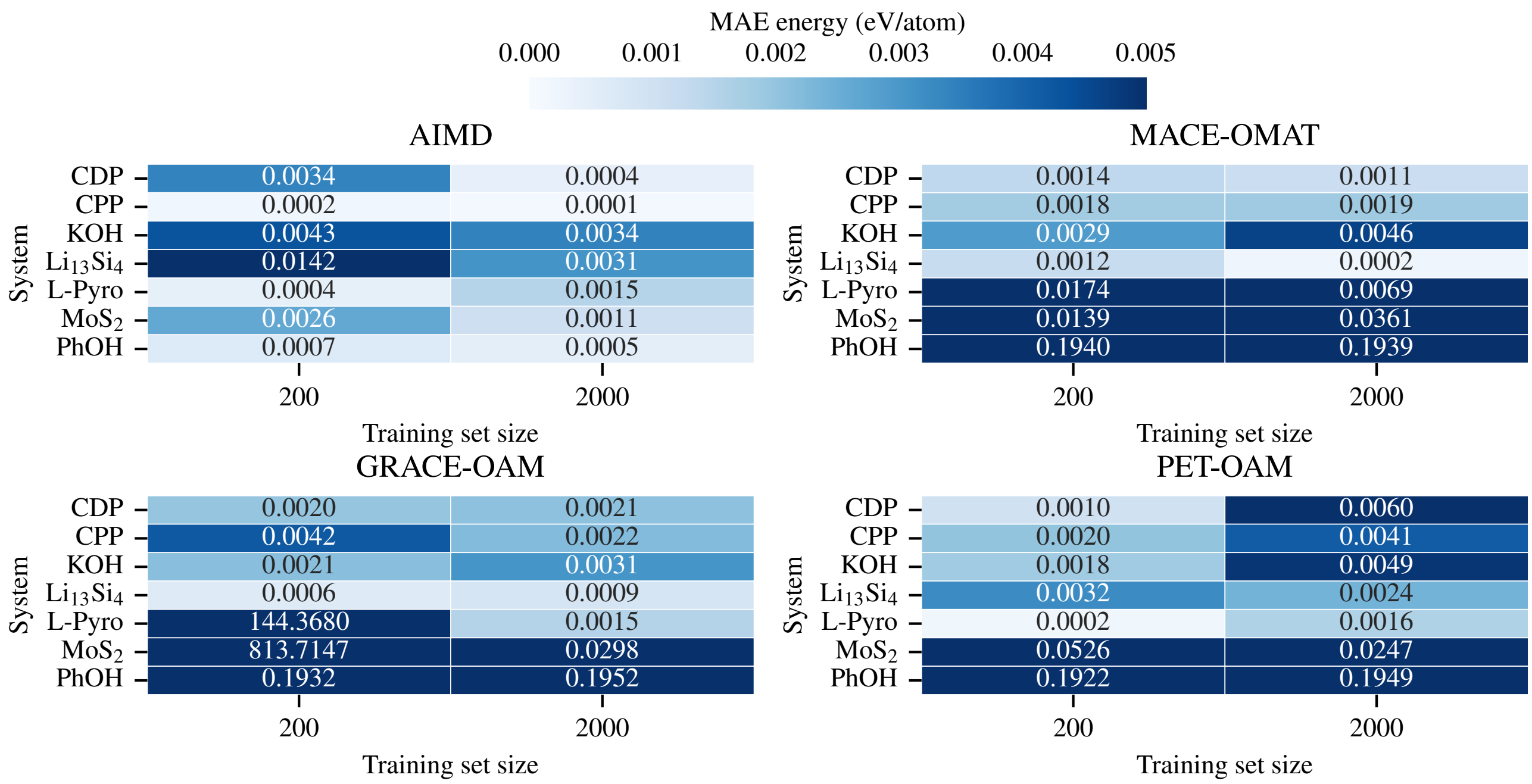

Supplementary Figure 12. **Fine-tuned MACE model energy errors**. Mean-absolute-error on the predicted energies of the fine-tuned MACE-MP-0 universal MLIPs (different universal dataset, 2000 frames) on the different systems: CsH_2PO_4 (CDP) and $Cs_7(H_4PO_4)(H_2PO_4)_8$ (CPP), aqueous KOH solution, $Li_{13}Si_4$, MoS_2, phenol in water and L-pyroglutamate-ammonium (L-Pyro).

SUPPLEMENTARY NOTE 14: FINE-TUNED MODEL (UNIVERSAL MODEL DATASET) - PREDICTION OF POTENTIAL ENERGY PROFILE FOR SULFUR-VACANCY JUMP IN MOS_2

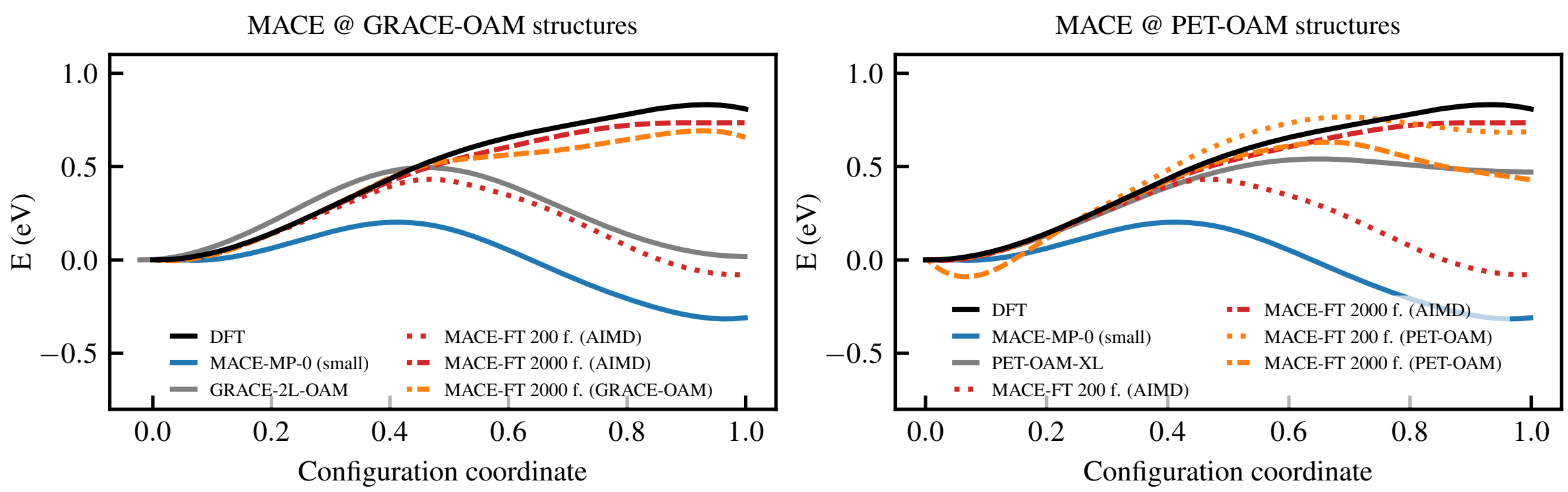

Supplementary Figure 13. **Energy profiles computed with fine-tuned models with variable universal model dataset**. Potential energy curves for a sulfur jump into a neighboring line of sulfur vacancies in MoS_2 via NEB calculations using fine-tuned MACE-MP-0 (small) universal models on DFT-recalculated universal model structures (orange lines) obtained with GRACE-2L-OAM (left) and PET-OAM-XL (right); and fine-tuned MACE-MP-0 (small) universal models on sub-sampled AIMD trajectories (red lines); with dataset sizes of 200 (dotted lines) and 2000 (dashed lines), MACE-MP-0 (small) (solid blue line) and the respective universal model (solid gray line) as well as the DFT reference profile (solid black line). The model fine-tuned on the dataset consisting out of 200 recalculated GRACE-2L-OAM data points is not able to compute converged structures for the NEB replicas.

SUPPLEMENTARY NOTE 15: MACE-MP-0 FINE-TUNED UNIVERSAL MODEL (MACE-OMAT DATASET) - PREDICTION OF PHYSICAL PROPERTIES

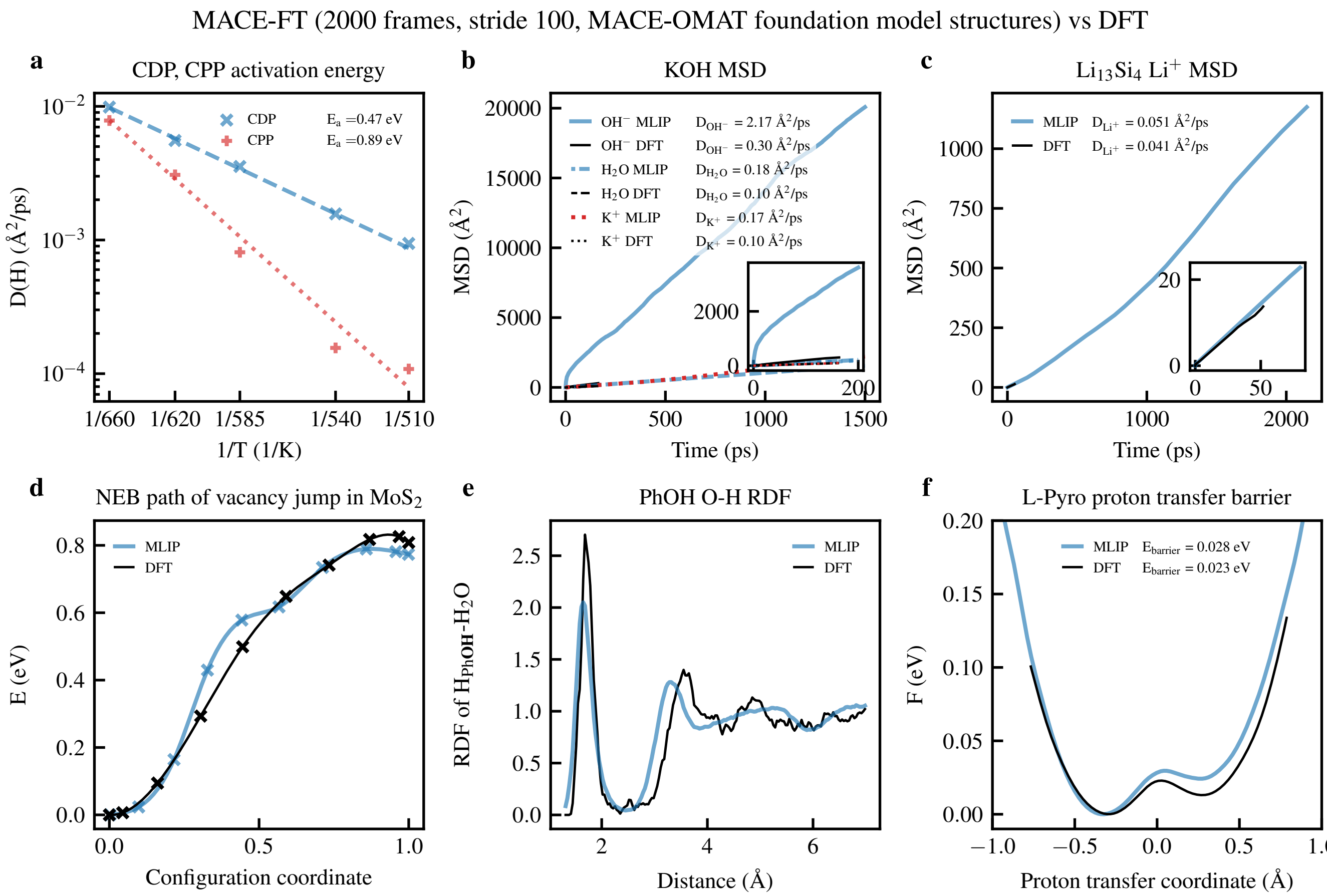

Supplementary Figure 14. **MACE-FT finetuned universal model on MACE-OMAT dataset**. Comparison of different physical properties obtained with first-principles methods and the fine-tuned universal models (MACE-OMAT data, 2000 frames, stride 3000): **a** CsH_2PO_4 (CDP) and $Cs_7(H_4PO_4)(H_2PO_4)_8$ (CPP) - activation energy of proton diffusion, **b** KOH - water, water molecule and hydroxide ion mean-squared displacements and diffusion coefficients, **c** $Li_{13}Si_4$ - lithium ion mean-squared displacements and diffusion coefficients, **d** MoS_2 - potential energy curves for a sulfur jump into a neighboring line of sulfur vacancies, **e** phenol in water - $(H_2O)\cdots O_{\text{Hydroxyl-Group}}$ radial distribution function and **f** L-pyroglutamate-ammonium (L-Pyro) - free energy profiles along the proton transfer coordinate.

SUPPLEMENTARY NOTE 16: MACE-MP-0 FINE-TUNED UNIVERSAL MODEL (PET-OAM DATASET) - PREDICTION OF PHYSICAL PROPERTIES

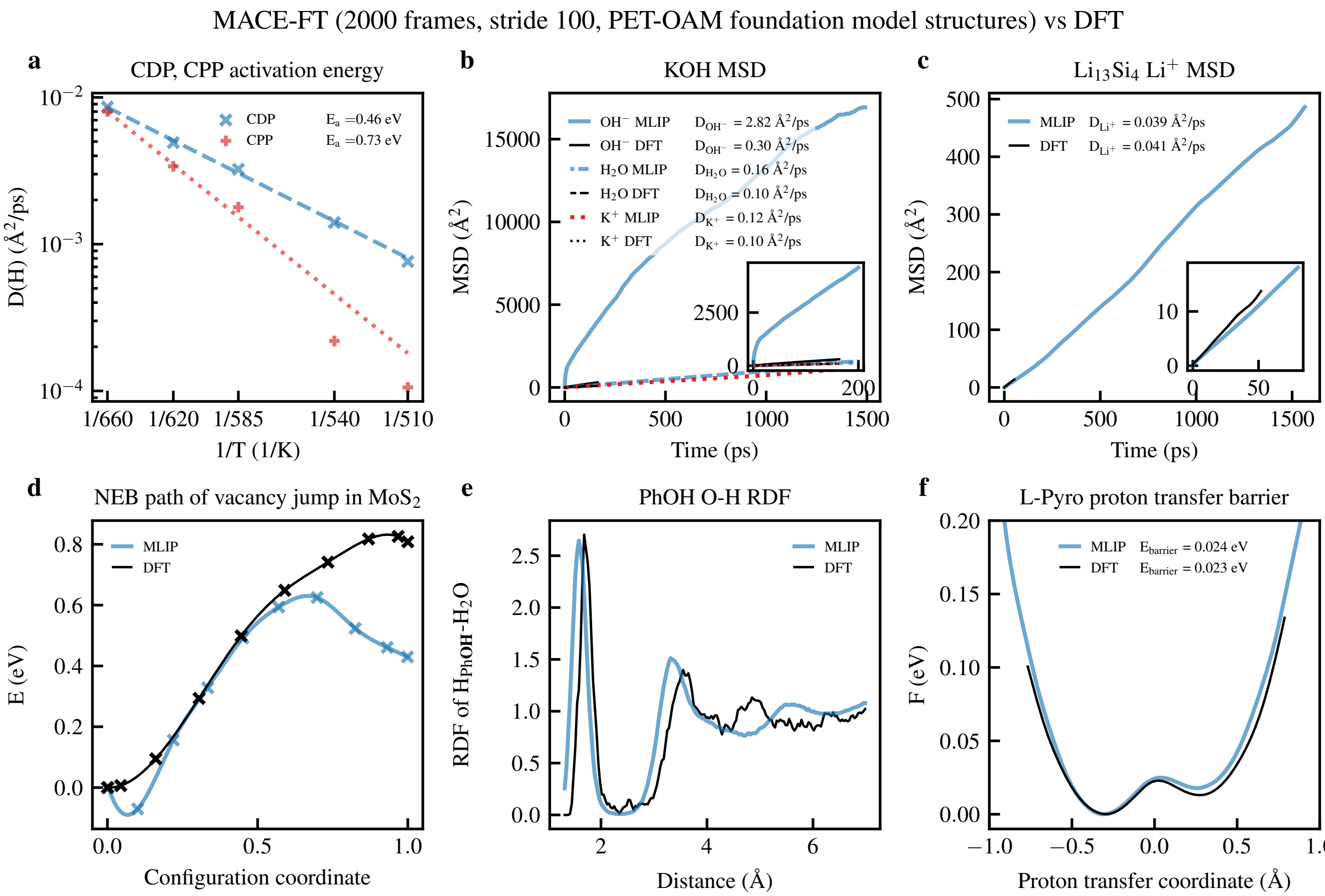

Supplementary Figure 15. **MACE finetuned universal model on PET-OAM dataset**. Comparison of different physical properties obtained with first-principles methods and the fine-tuned universal models (PET-OAM data, 2000 frames, stride 3000): **a** CsH_2PO_4 (CDP) and $Cs_7(H_4PO_4)(H_2PO_4)_8$ (CPP) - activation energy of proton diffusion, **b** KOH - water, water molecule and hydroxide ion mean-squared displacements and diffusion coefficients, **c** $Li_{13}Si_4$ - lithium ion mean-squared displacements and diffusion coefficients, **d** MoS_2 - potential energy curves for a sulfur jump into a neighboring line of sulfur vacancies, **e** phenol in water - $(H_2O)\cdots O_{\text{Hydroxyl-Group}}$ radial distribution function and **f** L-pyroglutamate-ammonium (L-Pyro) - free energy profiles along the proton transfer coordinate.

SUPPLEMENTARY NOTE 17: MACE-MP-0 FINE-TUNED UNIVERSAL MODEL (AIMD DATASET) - PREDICTION OF PHYSICAL PROPERTIES

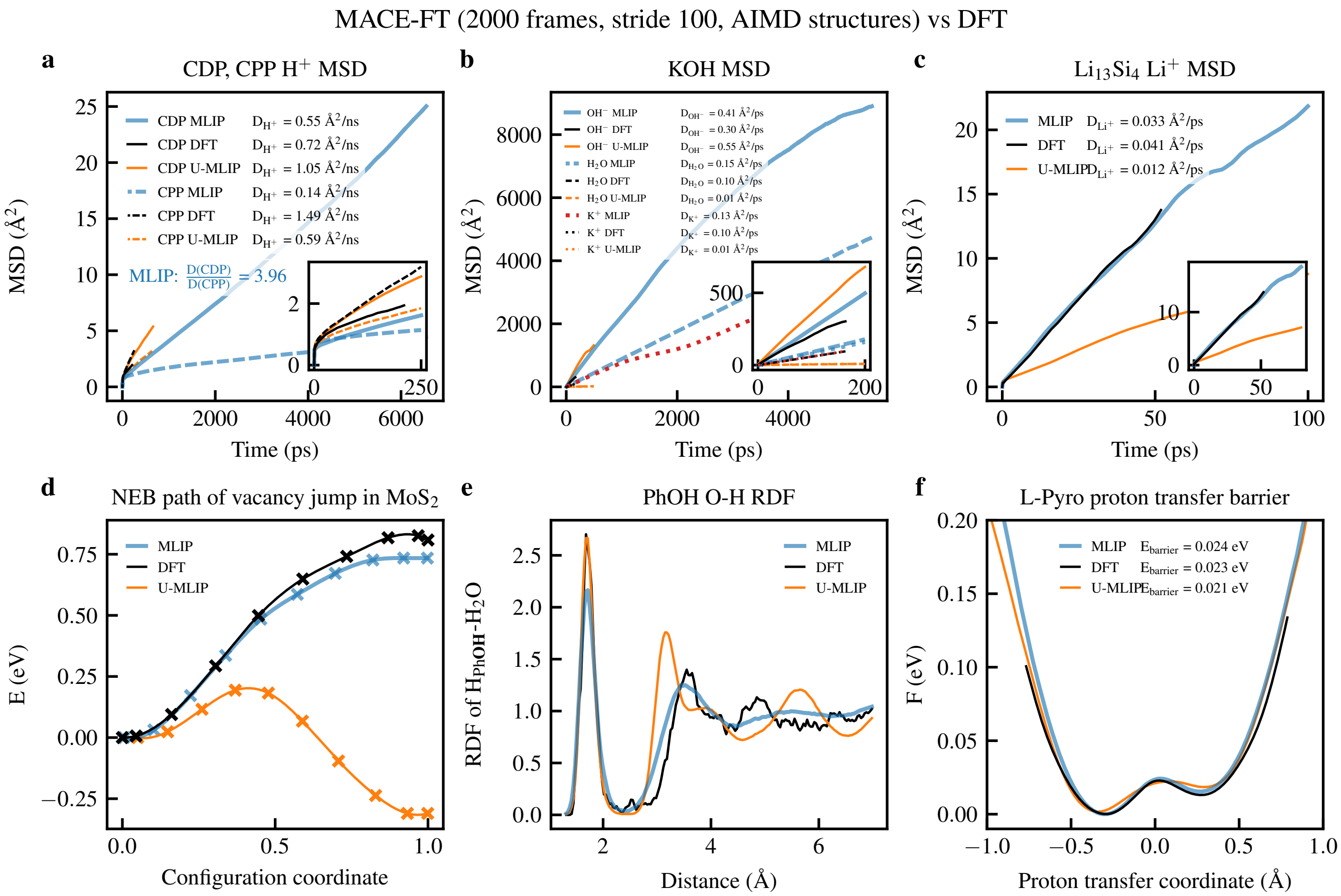

Supplementary Figure 16. **MACE-MP-0 finetuned universal model on AIMD dataset**. Comparison of different physical properties obtained with first-principles methods (black lines) and universal model MACE-MP-0 (orange lines) and the fine-tuned universal models (blue line, AIMD dataset, 2000 frames, stride 100): **a** CsH_2PO_4 (CDP) and $Cs_7(H_4PO_4)(H_2PO_4)_8$ (CPP) - activation energy of proton diffusion, **b** KOH - water, water molecule and hydroxide ion mean-squared displacements and diffusion coefficients, **c** $Li_{13}Si_4$ - lithium ion mean-squared displacements and diffusion coefficients, **d** MoS_2 - potential energy curves for a sulfur jump into a neighboring line of sulfur vacancies, **e** phenol in water - $(H_2O)\cdots O_{\text{Hydroxyl-Group}}$ radial distribution function and **f** L-pyroglutamate-ammonium (L-Pyro) - free energy profiles along the proton transfer coordinate.

SUPPLEMENTARY NOTE 18: FINE-TUNED MODEL PERFORMANCE - FORCE ERRORS - MLIPS VS. SYSTEMS VS. DATASET SIZE (MACE-OMAT UNIVERSAL MODEL DATASET)

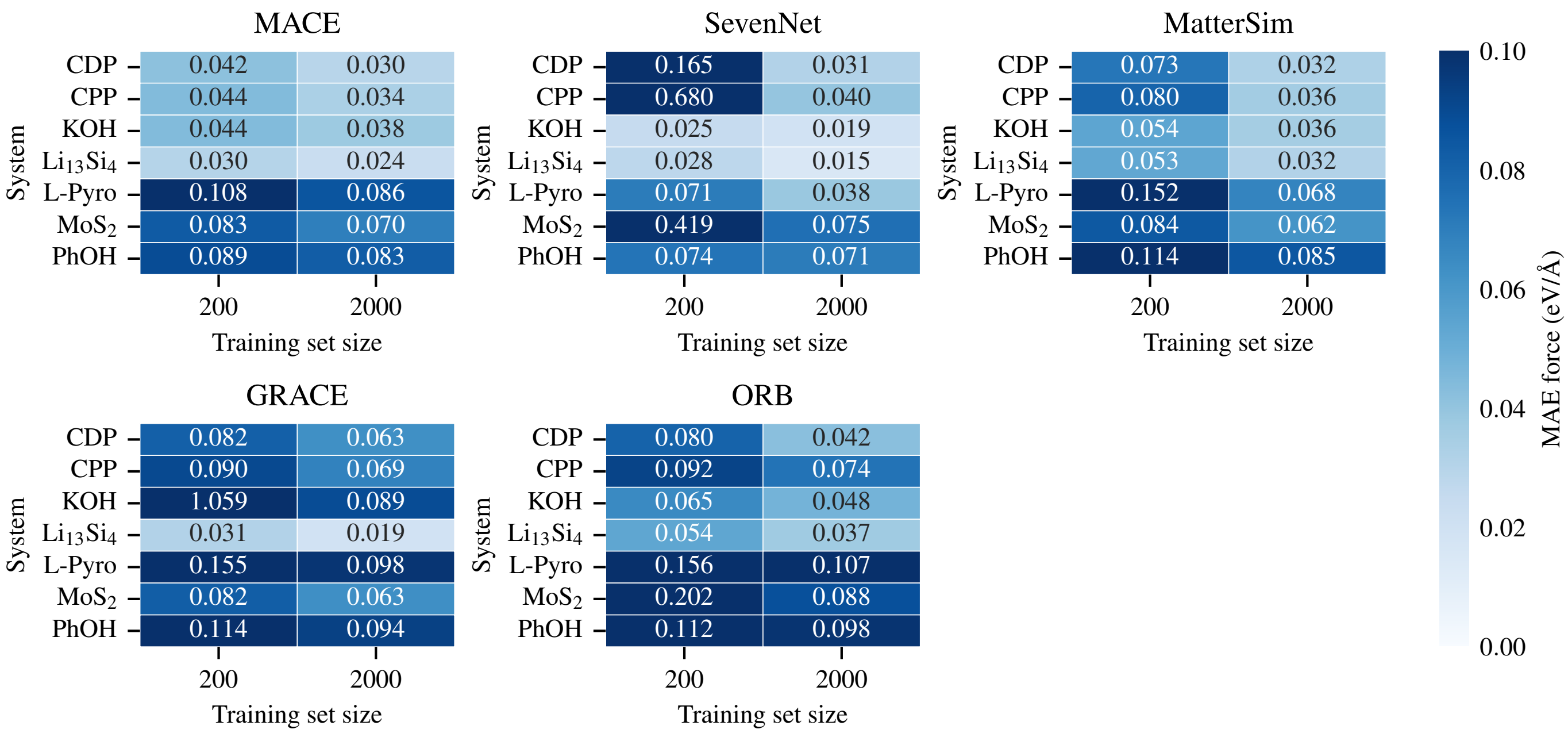

Supplementary Figure 17. **Fine-tuned model force errors**. Mean-absolute-error on the predicted forces of the different fine-tuned MLIPs (same MACE-OMAT-0 (medium) universal dataset, 2000 frames) on the different systems: CsH_2PO_4 (CDP) and $Cs_7(H_4PO_4)(H_2PO_4)_8$ (CPP), aqueous KOH solution, $Li_{13}Si_4$, MoS_2, phenol in water and L-pyroglutamate-ammonium (L-Pyro).

SUPPLEMENTARY NOTE 19: FINE-TUNED MODEL PERFORMANCE - ENERGY ERRORS - MLIPS VS. SYSTEMS VS. DATASET SIZE (MACE-OMAT UNIVERSAL MODEL DATASET)

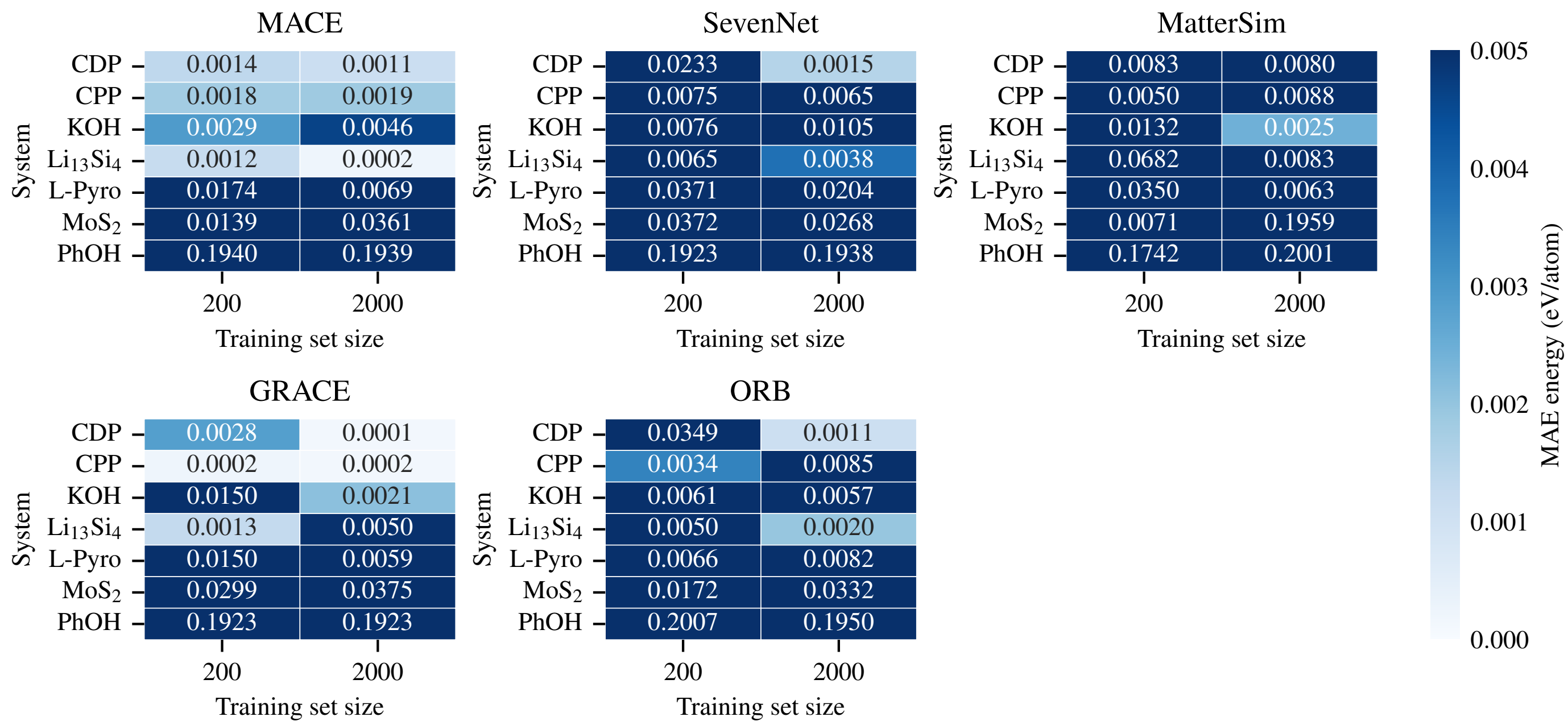

Supplementary Figure 18. **Fine-tuned model energy errors**. Mean-absolute-error on the predicted energies of the different fine-tuned MLIPs (same MACE-OMAT-0 (medium) universal dataset, 2000 frames) on the different systems: CsH_2PO_4 (CDP) and $Cs_7(H_4PO_4)(H_2PO_4)_8$ (CPP), aqueous KOH solution, $Li_{13}Si_4$, MoS_2, phenol in water and L-pyroglutamate-ammonium (L-Pyro).

SUPPLEMENTARY NOTE 20: FINE-TUNED MODEL (MACE-OMAT-0 MODEL DATASET) - PREDICTION OF POTENTIAL ENERGY PROFILE FOR SULFUR-VACANCY JUMP IN MoS_2

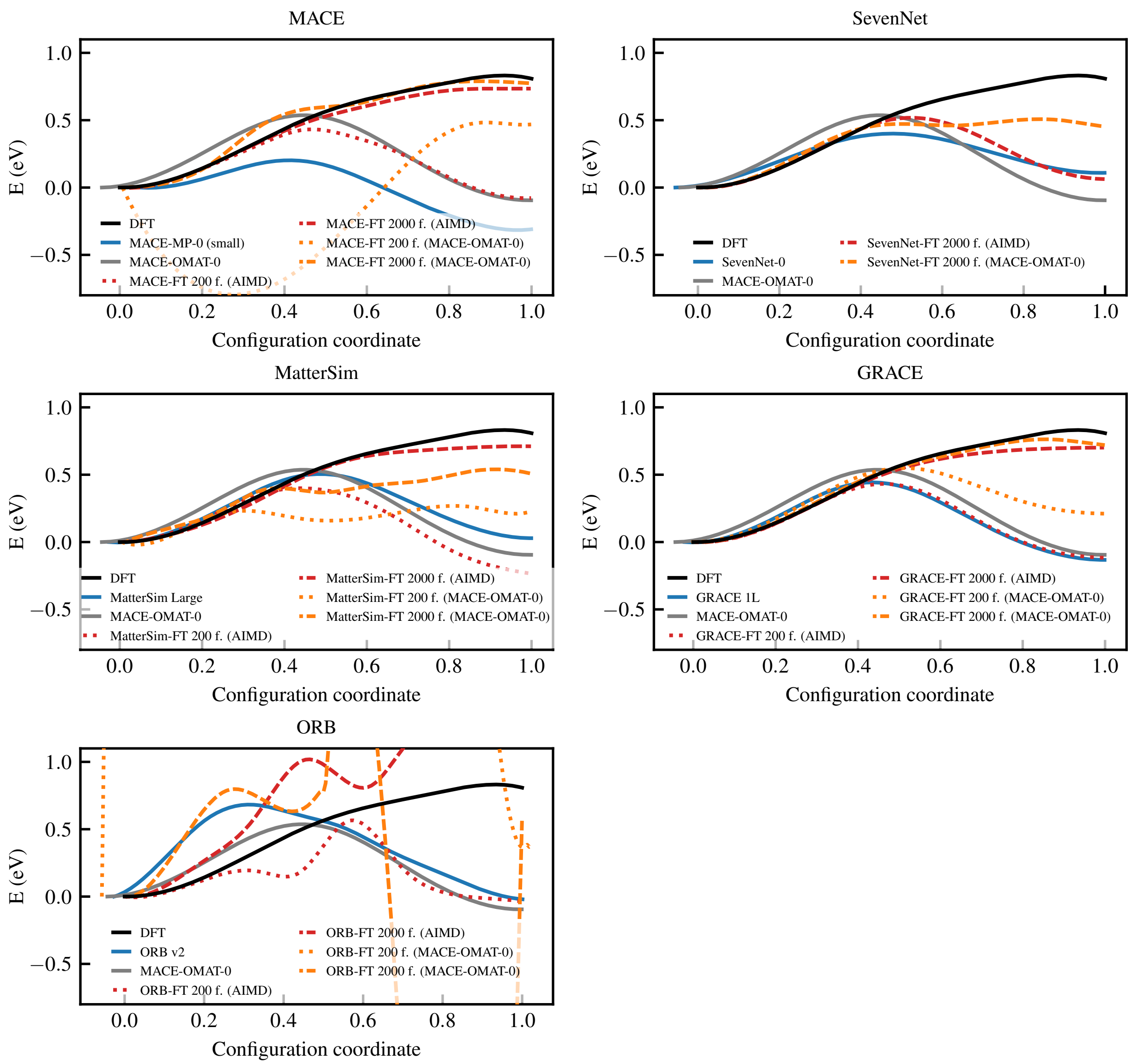

Supplementary Figure 19. **Energy profiles computed with fine-tuned models with variable universal model dataset**. Potential energy curves for a sulfur jump into a neighboring line of sulfur vacancies in MoS_2 via NEB calculations using fine-tuned MACE-MP-0 (small), SevenNet-0, MatterSim-v1.0.0-5M, GRACE-1L-OAM and ORB-v2 universal models on DFT-recalculated universal model structures (orange lines) obtained with MACE-OMAT-0 (medium); and fine-tuned universal models on sub-sampled AIMD trajectories (red lines); with dataset sizes of 200 (dotted lines) and 2000 (dashed lines), the respective universal model (solid blue line) and MACE-OMAT-0 (medium)(solid gray line) as well as the DFT reference profile (solid black line). The universal SevenNet-0 models fine-tuned on the datasets consisting out of 200 data points sub-sampled from an AIMD trajectories and 200 recalculated MACE-OMAT-0 (medium) data points are not able to compute converged structures for the NEB replicas.

SUPPLEMENTARY NOTE 21: SEVENNET-0 FINE-TUNED UNIVERSAL MODEL (MACE-OMAT DATASET) - PREDICTION OF PHYSICAL PROPERTIES

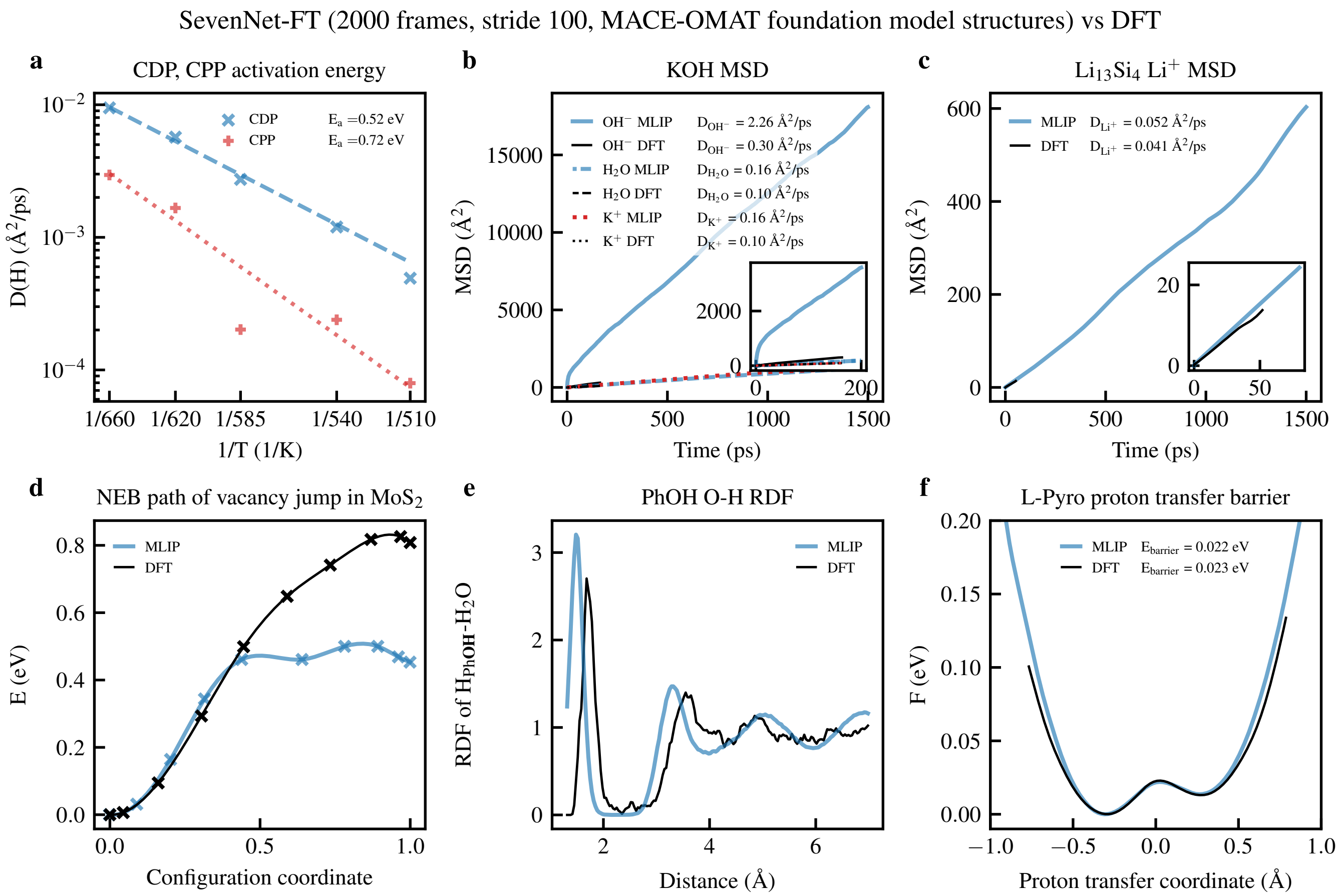

Supplementary Figure 20. **SevenNet-FT finetuned universal model on MACE-OMAT dataset**. Comparison of different physical properties obtained with first-principles methods and the fine-tuned universal models (MACE-OMAT data, 2000 frames, stride 3000): **a** CsH_2PO_4 (CDP) and $Cs_7(H_4PO_4)(H_2PO_4)_8$ (CPP) - activation energy of proton diffusion, **b** KOH - water, water molecule and hydroxide ion mean-squared displacements and diffusion coefficients, **c** $Li_{13}Si_4$ - lithium ion mean-squared displacements and diffusion coefficients, **d** MoS_2 - potential energy curves for a sulfur jump into a neighboring line of sulfur vacancies, **e** phenol in water - $(H_2O)\cdots O_{\text{Hydroxyl-Group}}$ radial distribution function and **f** L-pyroglutamate-ammonium (L-Pyro) - free energy profiles along the proton transfer coordinate.

SUPPLEMENTARY NOTE 22: MATTERSIM-LARGE FINE-TUNED UNIVERSAL MODEL (MACE-OMAT DATASET) - PREDICTION OF PHYSICAL PROPERTIES

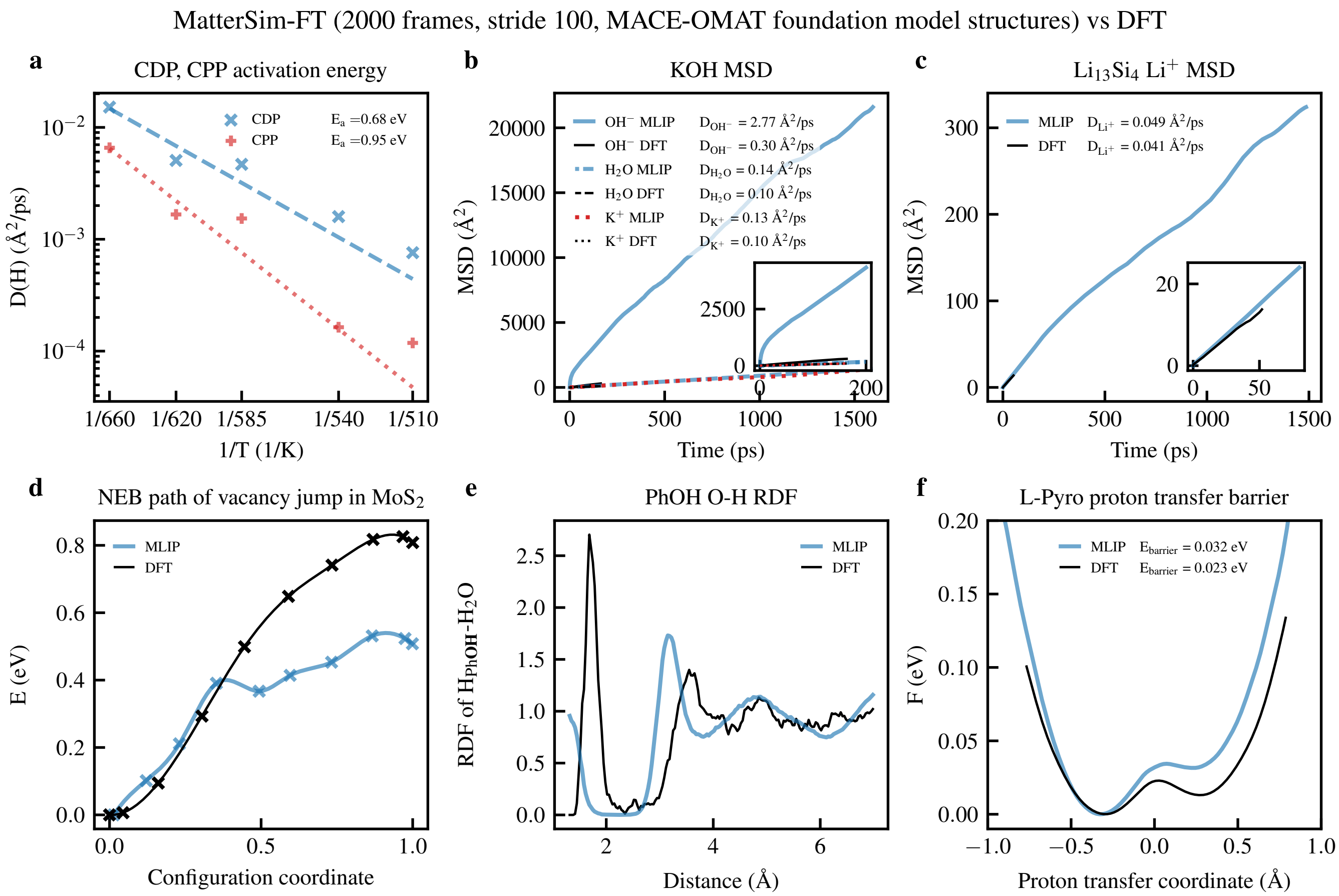

Supplementary Figure 21. **MatterSim-FT finetuned universal model on MACE-OMAT dataset**. Comparison of different physical properties obtained with first-principles methods and the fine-tuned universal models (MACE-OMAT data, 2000 frames, stride 3000): **a** CsH_2PO_4 (CDP) and $Cs_7(H_4PO_4)(H_2PO_4)_8$ (CPP) - activation energy of proton diffusion, **b** KOH - water, water molecule and hydroxide ion mean-squared displacements and diffusion coefficients, **c** $Li_{13}Si_4$ - lithium ion mean-squared displacements and diffusion coefficients, **d** MoS_2 - potential energy curves for a sulfur jump into a neighboring line of sulfur vacancies, **e** phenol in water - $(H_2O)\cdots O_{\text{Hydroxyl-Group}}$ radial distribution function and **f** L-pyroglutamate-ammonium (L-Pyro) - free energy profiles along the proton transfer coordinate.

SUPPLEMENTARY NOTE 23: TRAINED MACE MODEL PERFORMANCE - FORCE ERRORS - DATASET GENERATING UNIVERSAL MODEL VS. SYSTEMS VS. DATASET SIZE (UNIVERSAL MODEL DATASET)

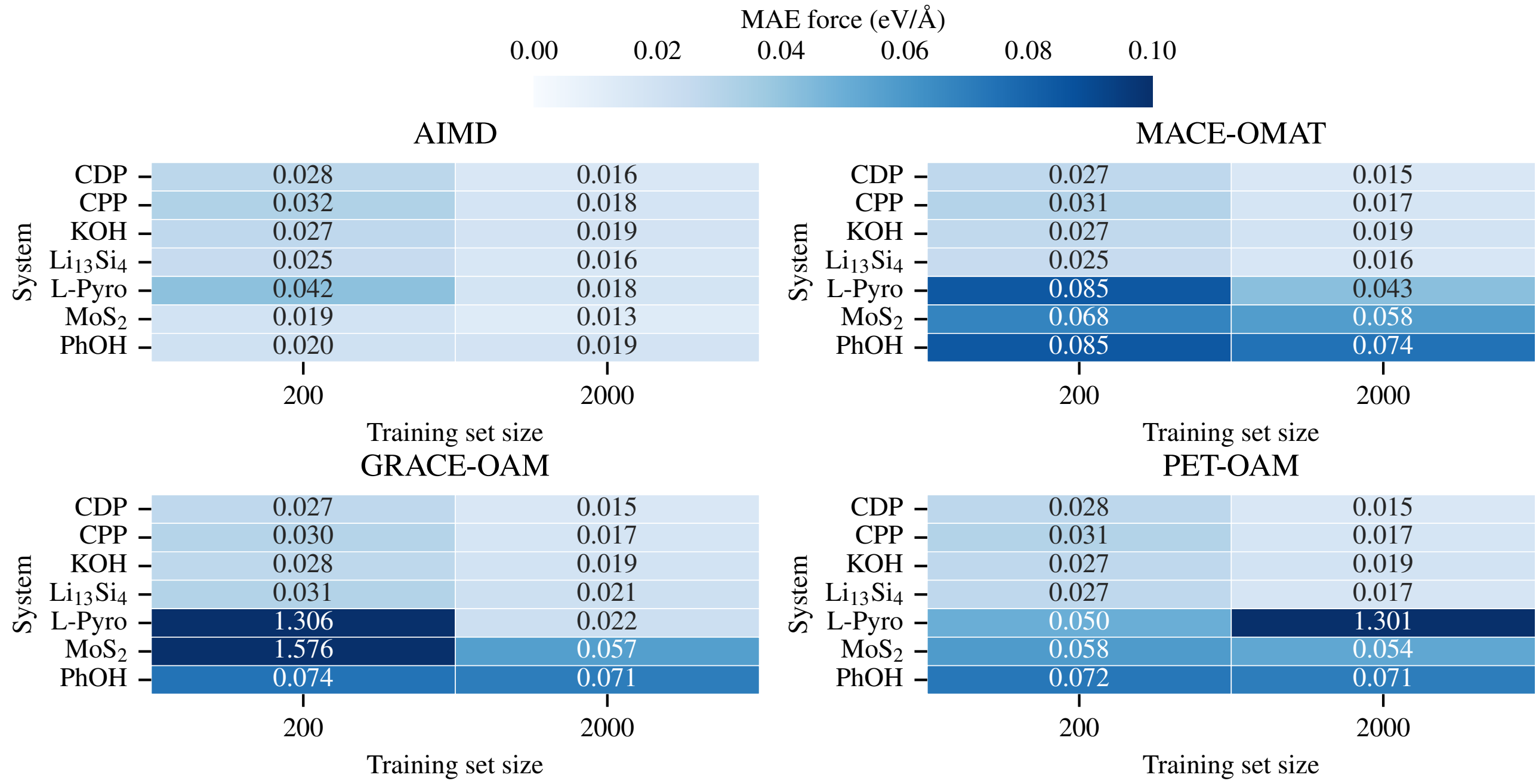

Supplementary Figure 22. **Trained-from-scratch MACE model force errors**. Mean-absolute-error on the predicted forces of the trained-from-scratch MACE MLIPs (different universal dataset, 2000 frames) on the different systems: CsH_2PO_4 (CDP) and $Cs_7(H_4PO_4)(H_2PO_4)_8$ (CPP), aqueous KOH solution, $Li_{13}Si_4$, MoS_2, phenol in water and L-pyroglutamate-ammonium (L-Pyro).

SUPPLEMENTARY NOTE 24: TRAINED MACE MODEL PERFORMANCE - ENERGY ERRORS - DATASET GENERATING UNIVERSAL MODEL VS. SYSTEMS VS. DATASET SIZE (UNIVERSAL MODEL DATASET)

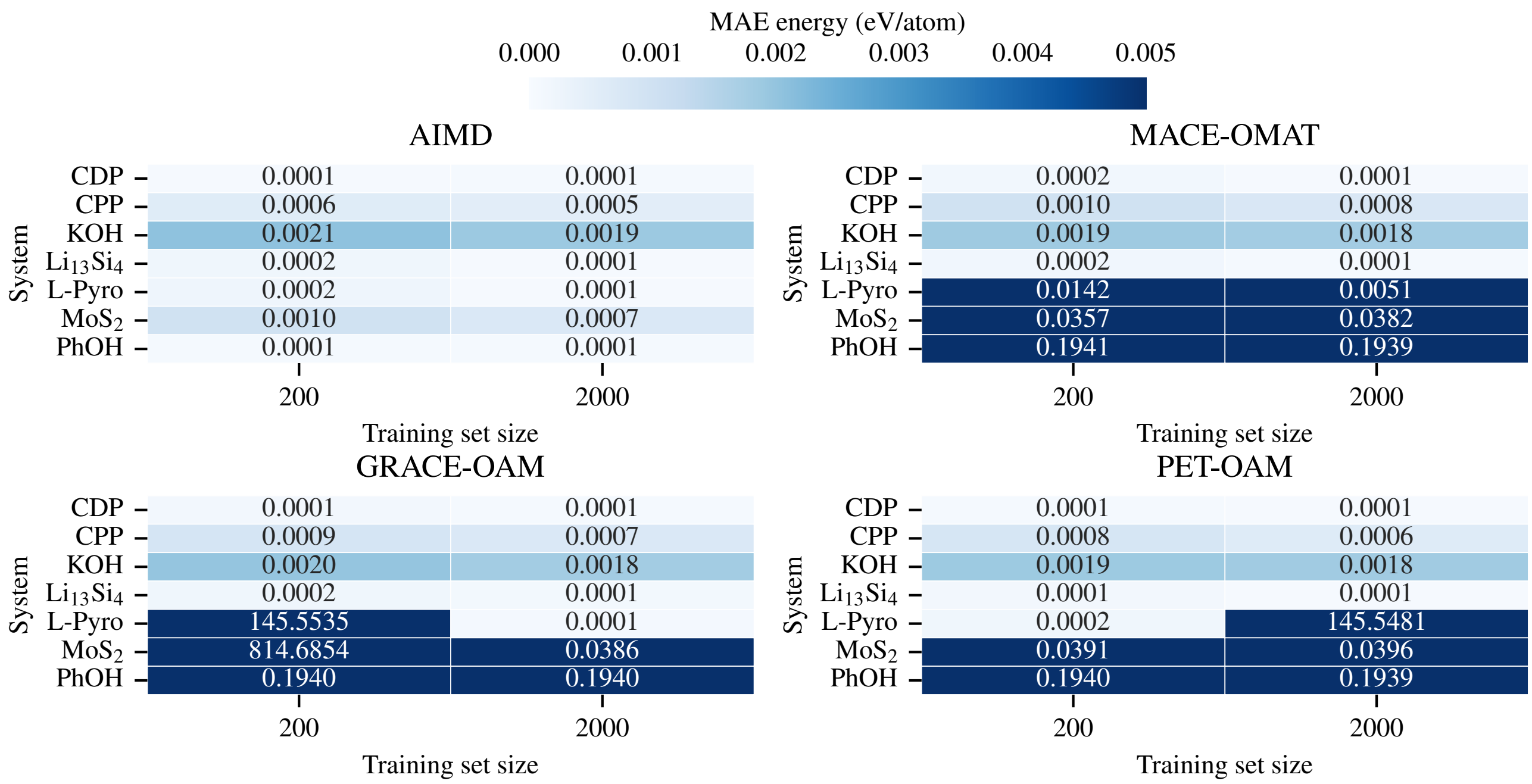

Supplementary Figure 23. **Trained-from-scratch MACE model energy errors**. Mean-absolute-error on the predicted energies of the trained-from-scratch MACE MLIPs (different universal dataset, 2000 frames) on the different systems: CsH_2PO_4 (CDP) and $Cs_7(H_4PO_4)(H_2PO_4)_8$ (CPP), aqueous KOH solution, $Li_{13}Si_4$, MoS_2, phenol in water and L-pyroglutamate-ammonium (L-Pyro).

SUPPLEMENTARY NOTE 25: TRAINED-FROM-SCRATCH MODEL (UNIVERSAL MODEL DATASET) - PREDICTION OF POTENTIAL ENERGY PROFILE FOR SULFUR-VACANCY JUMP IN MOS_2

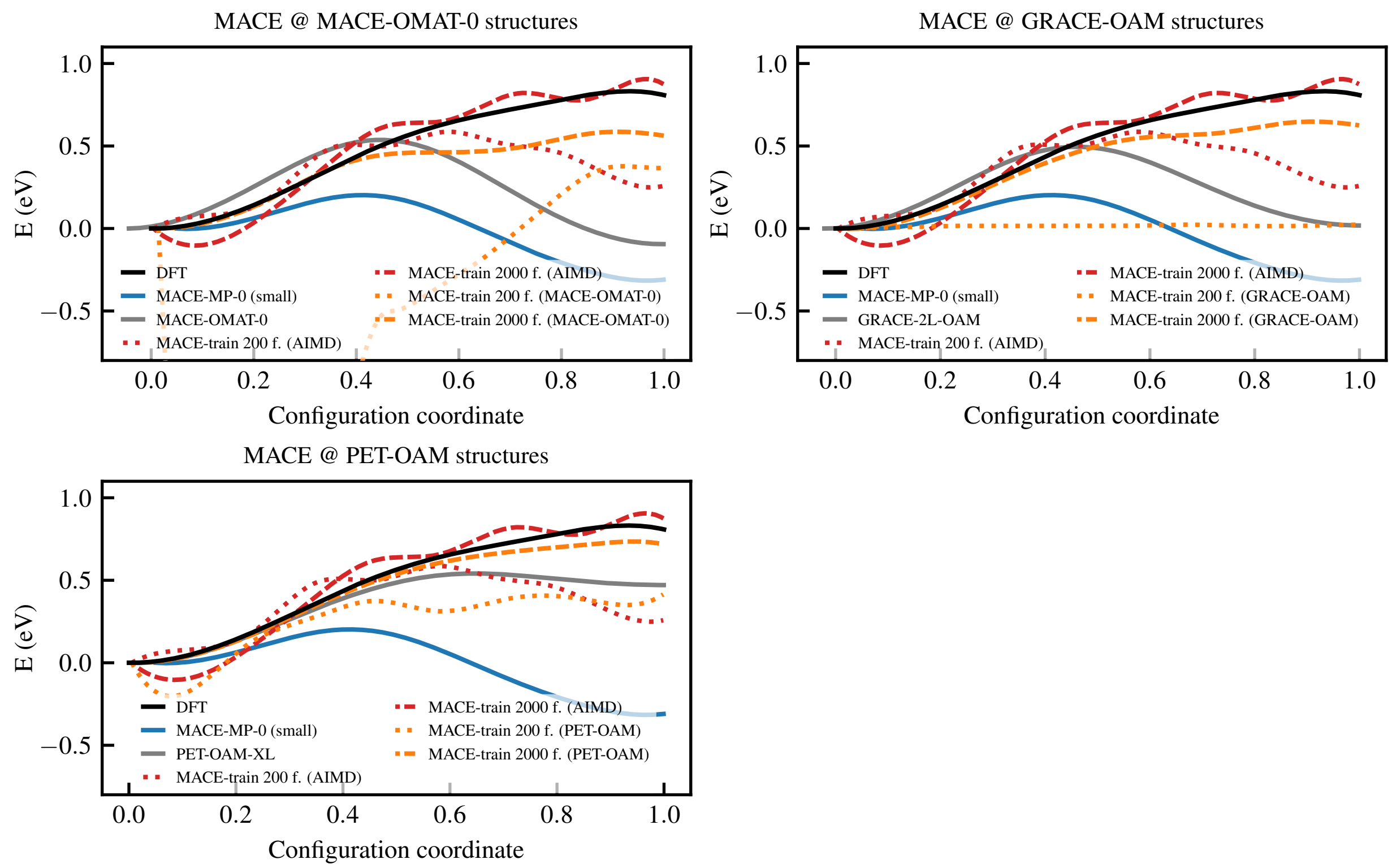

Supplementary Figure 24. **Energy profiles computed with trained-from-scratch models with variable universal model dataset**. Potential energy curves for a sulfur jump into a neighboring line of sulfur vacancies in MoS_2 via NEB calculations using trained-from-scratch MACE models on DFT-recalculated universal model structures (orange lines) obtained with GRACE-2L-OAM (left) and PET-OAM-XL (right) and trained-from-scratch MACE models on sub-sampled AIMD trajectories (red lines); with dataset sizes of 200 (dotted lines) and 2000 (dashed lines), MACE-MP-0 (small) (solid blue line) and the respective universal model (solid gray line) as well as the DFT reference profile (solid black line).

SUPPLEMENTARY NOTE 26: COMPARING ITERATIVE TRAINING AGAINST SINGLE TIME FINE-TUNING - PREDICTION OF POTENTIAL ENERGY PROFILE FOR SULFUR-VACANCY JUMP IN MOS_2

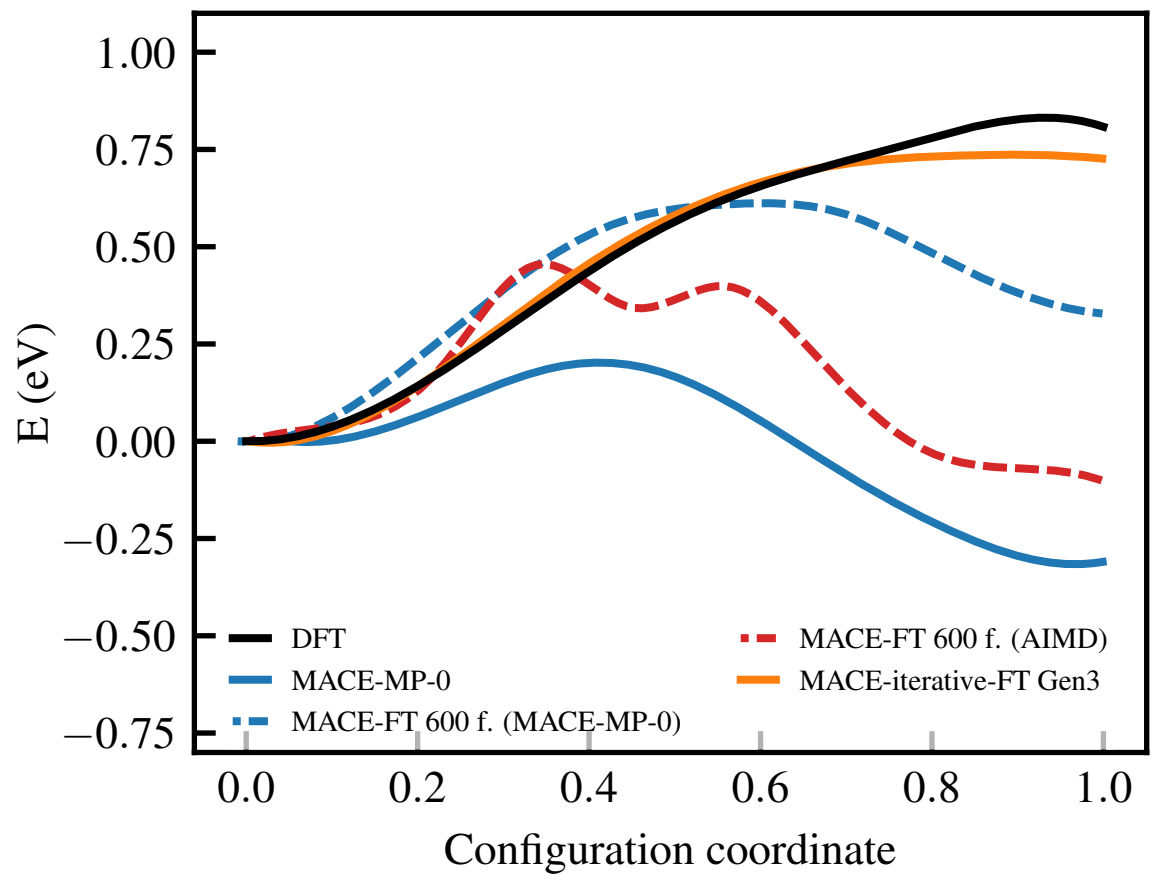

Supplementary Figure 25. **Energy profiles computed with iterative-fine-tuned and singular fine-tuned models**. Potential energy curves for a sulfur jump into a neighboring line of sulfur vacancies in MoS_2 via NEB calculations using datasets build up from: 600 data points from a sub-sampled MACE-MP-0 trajectory (dashed blue line) and 600 data points from a sub-sampled AIMD trajectory (dashed red line), the third generation of the iterative training approach shown in main text in Figure 5e (solid orange line), the respective universal model (solid blue line) as well as the DFT reference profile (solid black line).

SUPPLEMENTARY NOTE 27: ITERATIVE TRAINING ADDITIONAL MODEL GENERATIONS

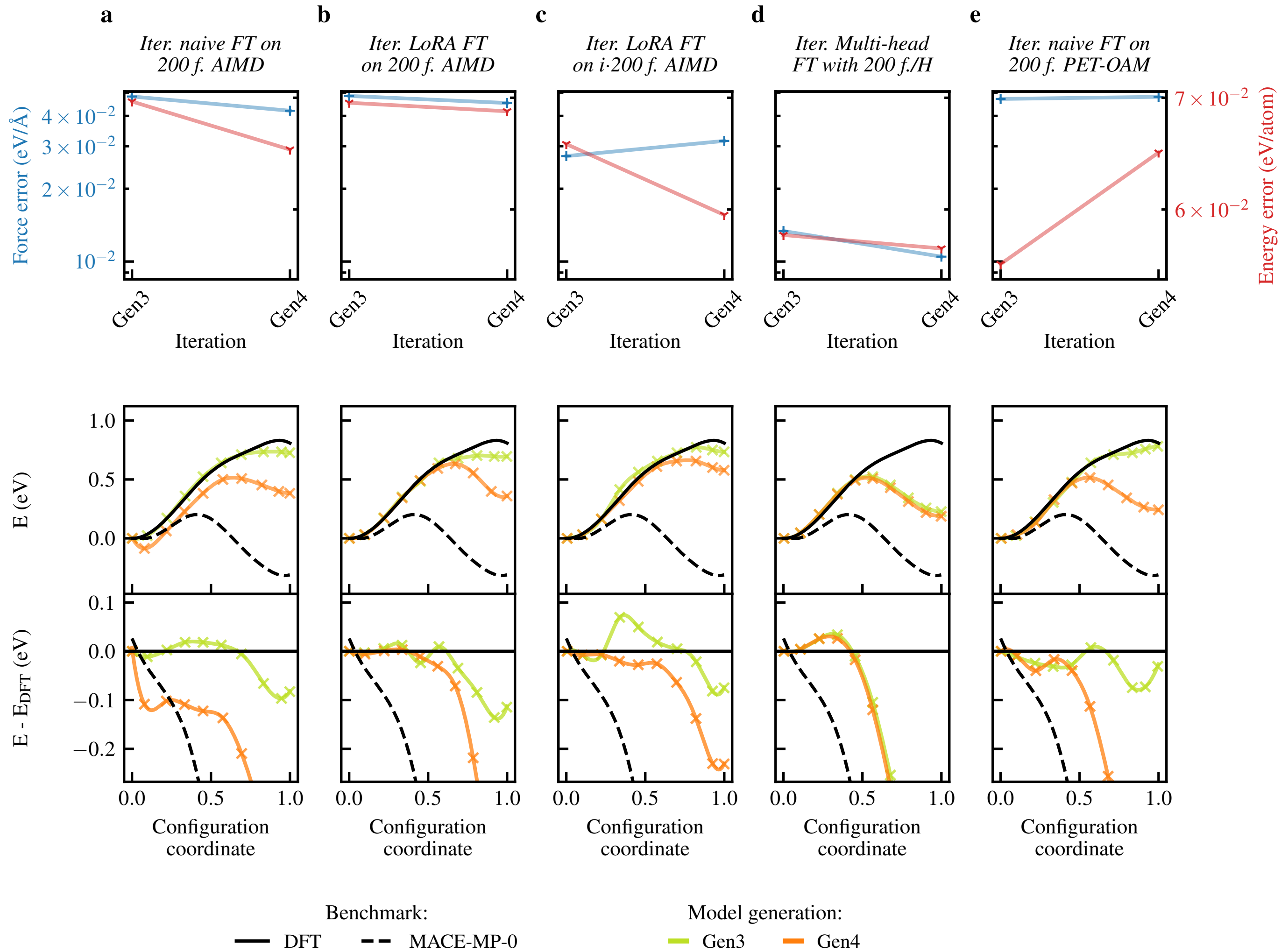

Supplementary Figure 26. **Iterative model training generations**. Iterative training models of generation 3 and 4. The models of generation 4 tend to perform worse then the previous generation because of the bad training data composition.

SUPPLEMENTARY NOTE 28: COMPARING ITERATIVE TRAINING DATASET COMPOSITION

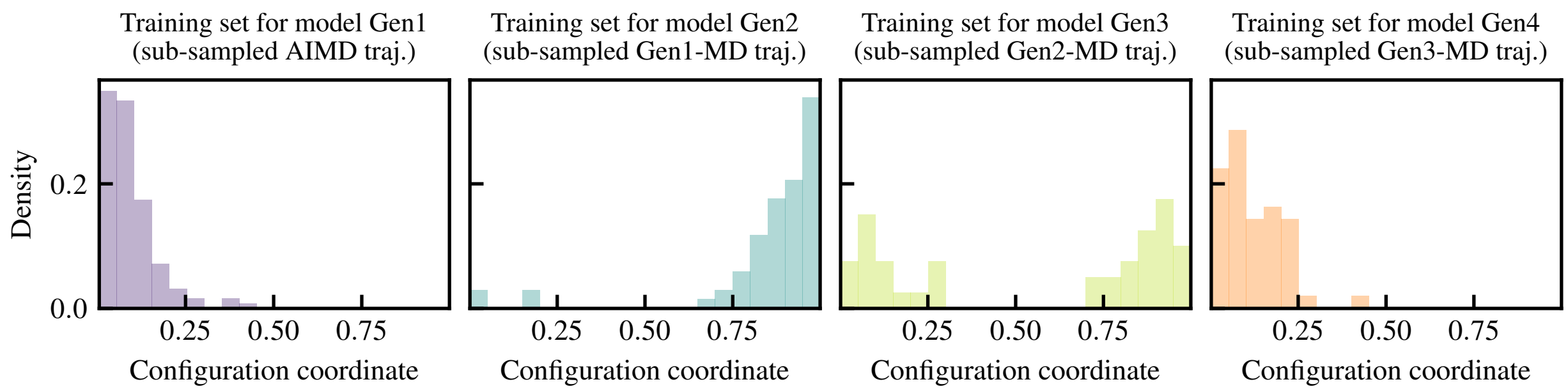

Supplementary Figure 27. **Dataset composition for different model generation**. Training set composition with respect to the configuration coordinate of the sulfur-vacancy jump of the different model generations.

SUPPLEMENTARY NOTE 29: FINE-TUNED MODEL PERFORMANCE WITH RESPECT TO THE CONFIGURATION SPACE SAMPLING METHOD- NEB ENERGY PROFILES WITH UNCERTAINTY ESTIMATION

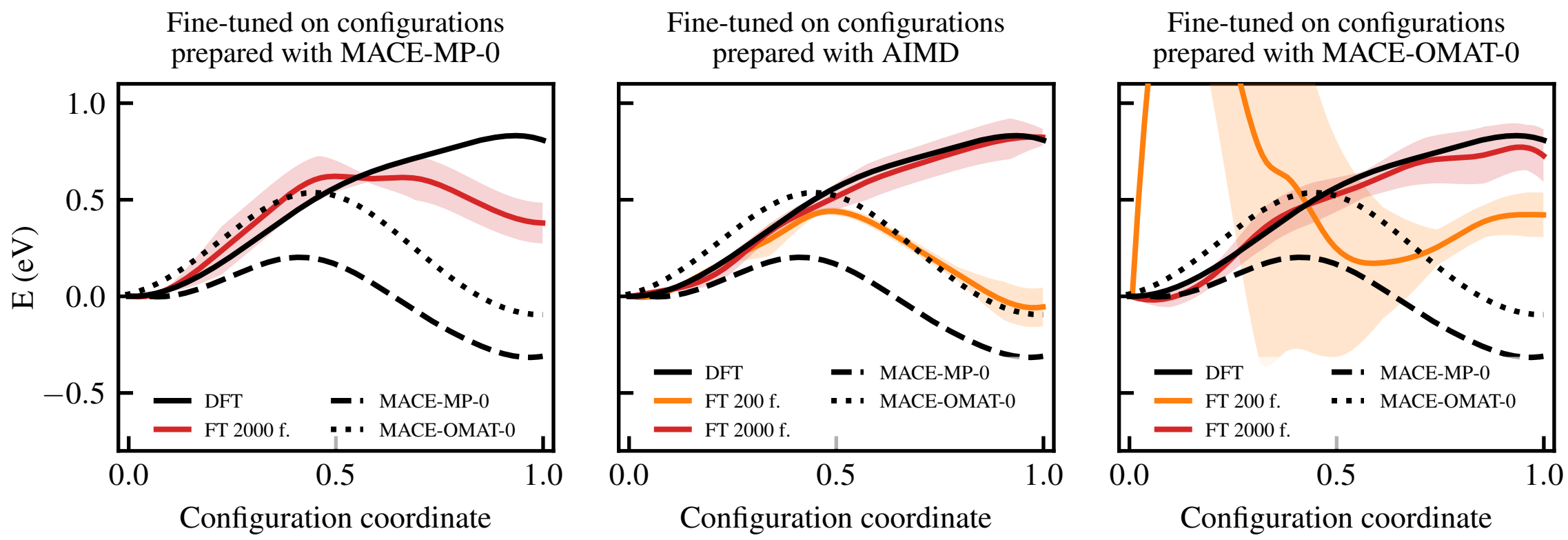

Supplementary Figure 28. **MoS_2 NEB performance of fine-tuned models trained on different datasets, including uncertainty estimation.** Potential energy profiles for sulfur migration into a neighboring sulfur-vacancy line, obtained from nudged elastic band calculations using models fine-tuned on 200 (orange) and 2,000 (red) DFT-relabeled structures. The training datasets were generated by subsampling trajectories produced with the universal MACE-MP-0 model (left), AIMD (center), or the universal MACE-OMAT-0 model (right). Results are compared with the universal MACE-MP-0 (small) model (black dashed line), the universal MACE-OMAT-0 (medium) model (black dotted line), and the DFT reference (black solid line). Uncertainties correspond to the standard deviation of the mean energy predicted by an ensemble of five independently fine-tuned models trained on the same dataset using different seeds (1, 2, 3, 4, 5). This figure presents the same data as Figure 2e in the main text, augmented with uncertainty estimates.

SUPPLEMENTARY NOTE 30: ITERATIVE FINE-TUNING PERFORMANCE WITH RESPECT TO THE CONFIGURATION SPACE SAMPLING METHOD - NEB ENERGY PROFILES WITH UNCERTAINTY ESTIMATION

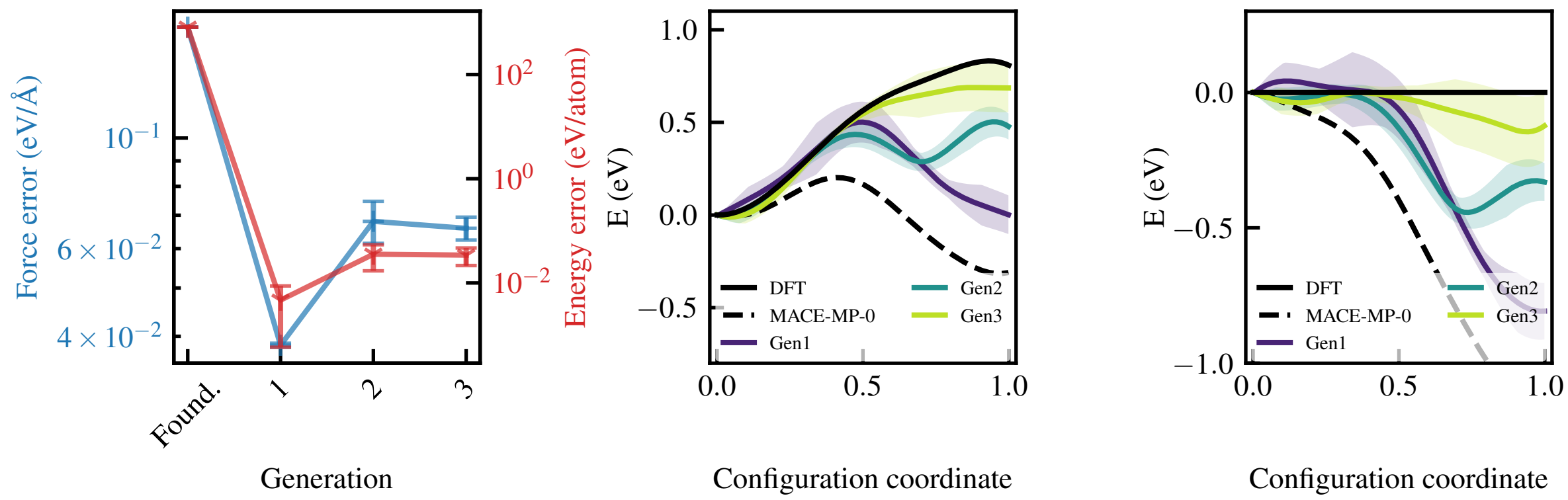

Supplementary Figure 29. **Evaluation of three generations of iteratively fine-tuned MACE-MP-0 models for MoS_2 with uncertainty estimation.** Force (blue) and energy (red) mean absolute errors (MAEs) on the test set are shown on the left for the universal MACE-MP-0 model (orange) and the three generations of the iterative fine-tuning workflow, starting from an initial training set of 200 structures subsampled from an AIMD trajectory (Gen1, violet; Gen2, turquoise; Gen3, green). The center panel compares the potential energy profiles for sulfur migration into a neighboring sulfur-vacancy line, obtained from nudged elastic band calculations using the iteratively fine-tuned models (Gen1-Gen3), the universal MACE-MP-0 (small) model (black dashed line), the universal MACE-OMAT-0 (medium) model (black dotted line), and the DFT reference (black solid line). The right panel shows the deviation of each model from the DFT reference profile. Uncertainties are reported as the standard deviation of the mean energy predicted by an ensemble of five independently fine-tuned models trained on the same dataset using different seeds (1, 2, 3, 4, 5). This figure presents the same data as Figure 3a in the main text, augmented with uncertainty estimates.

* jonas.haenseroth@tu-ilmenau.de

[1] I. Batatia, P. Benner, Y. Chiang, A. M. Elena, D. P. Kovács, J. Riebesell, X. R. Advincula, M. Asta, M. Avaylon, W. J. Baldwin, F. Berger, N. Bernstein, A. Bhowmik, F. Bigi, S. M. Blau, V. Cărare, M. Ceriotti, S. Chong, J. P. Darby, S. De, F. Della Pia, V. L. Deringer, R. Elijošius, Z. El-Machachi, E. Fako, F. Falcioni, A. C. Ferrari, J. L. A. Gardner, M. J. Gawkowski, A. Genreith-Schriever, J. George, R. E. A. Goodall, J. Grandel, C. P. Grey, P. Grigorev, S. Han, W. Handley, H. H. Heenen, K. Hermansson, C. H. Ho, S. Hofmann, C. Holm, J. Jaafar, K. S. Jakob, H. Jung, V. Kapil, A. D. Kaplan, N. Karimitari, J. R. Kermode, P. Kourtis, N. Kroupa, J. Kullgren, M. C. Kuner, D. Kuryla, G. Liepuoniute, C. Lin, J. T. Margraf, I.-B. Magdău, A. Michaelides, J. H. Moore, A. A. Naik, S. P. Niblett, S. W. Norwood, N. O'Neill, C. Ortner, K. A. Persson, K. Reuter, A. S. Rosen, L. A. M. Rosset, L. L. Schaaf, C. Schran, B. X. Shi, E. Sivonxay, T. K. Stenczel, C. Sutton, V. Svahn, T. D. Swinburne, J. Tilly, C. van der Oord, S. Vargas, E. Varga-Umbrich, T. Vegge, M. Vondrák, Y. Wang, W. C. Witt, T. Wolf, F. Zills, and G. Csányi, A foundation model for atomistic materials chemistry, J. Chem. Phys. **163**, 184110 (2025).

[2] H. Yang, C. Hu, Y. Zhou, X. Liu, Y. Shi, J. Li, G. Li, Z. Chen, S. Chen, C. Zeni, *et al.*, Mattersim: A deep learning atomistic model across elements, temperatures and pressures, arXiv:2405.04967 (2024).

[3] Y. Park, J. Kim, S. Hwang, and S. Han, Scalable parallel algorithm for graph neural network interatomic potentials in molecular dynamics simulations, J. Chem. Theory Comput. **20**, 4857 (2024).

[4] Y. Lysogorskiy, A. Bochkarev, and R. Drautz, Graph atomic cluster expansion for foundational machine learning interatomic potentials, npj Comput. Mater. 10.1038/s41524-026-01979-1 (2026).

[5] M. Neumann, J. Gin, B. Rhodes, S. Bennett, Z. Li, H. Choubisa, A. Hussey, and J. Godwin, Orb: A fast, scalable neural network potential, arXiv:2410.22570 (2024).

[6] J. Hänseroth, A. Flötotto, M. N. Qaisrani, and C. Dreßler, Fine-tuning unifies foundational machine-learned interatomic potential architectures at ab initio accuracy, J. Phys. Chem. Lett. **17**, 3152 (2026).

[7] I. Batatia, C. Lin, J. Hart, E. Kasoar, A. M. Elena, S. W. Norwood, T. Wolf, and C. Gábor, Cross learning between electronic structure theories for unifying molecular, surface, and inorganic crystal foundation force fields, arXiv:2510.25380 (2025).

[8] A. Mazitov, F. Bigi, M. Kellner, P. Pegolo, D. Tisi, G. Fraux, S. Pozdnyakov, P. Loche, and M. Ceriotti, Pet-mad as a lightweight universal interatomic potential for advanced materials modeling, Nat. Commun. **16**, 10653 (2025).

[9] C. Malosso, F. Bigi, P. Pegolo, J. W. Abbott, P. Loche, M. Rossi, M. Ceriotti, and A. Mazitov, High-quality, high-information datasets for universal atomistic machine learning, arXiv:2603.02089 (2026).

[10] F. Bigi, P. Pegolo, A. Mazitov, J. Schmidt, and M. Ceriotti, Pushing the limits of unconstrained machine-learned interatomic potentials, Mach. Learn.: Sci. Technol. **7**, 035051 (2026).

[11] J. Schmidt, N. Hoffmann, H.-C. Wang, P. Borlido, P. J. M. A. Carriço, T. F. T. Cerqueira, S. Botti, and M. A. L. Marques, Machine-learning-assisted determination of the global zero-temperature phase diagram of materials, Adv. Mater. **35**, 2210788 (2023).

[12] L. Barros-Luque, M. Shuaibi, X. Fu, B. M. Wood, M. Dzamba, M. Gao, A. Rizvi, M. Uyttendaele, C. L. Zitnick, and Z. W. Ulissi, The Open Materials 2024 (OMat24) inorganic materials dataset and models, Nat. Comput. Sci. **6**, 642 (2026).

[13] A. Jain, S. P. Ong, G. Hautier, W. Chen, W. D. Richards, S. Dacek, S. Cholia, D. Gunter, D. Skinner, G. Ceder, and K. A. Persson, Commentary: The Materials Project: A materials genome approach to accelerating materials innovation, APL Mater. **1**, 011002 (2013).

[14] S. P. Ong, S. Cholia, A. Jain, M. Brafman, D. Gunter, G. Ceder, and K. A. Persson, The Materials Application Programming Interface (API): A simple, flexible and efficient API for materials data based on REpresentational State Transfer (REST) principles, Comput. Mater. Sci. **97**, 209 (2015).

[15] B. Deng, P. Zhong, K. Jun, J. Riebesell, K. Han, C. J. Bartel, and G. Ceder, Chgnet as a pretrained universal neural network potential for charge-informed atomistic modelling, Nat. Mach. Intell. **5**, 1031 (2023).

[16] D. S. Levine, M. Shuaibi, E. W. Clark Spotte-Smith, M. G. Taylor, M. R. Hasyim, K. Michel, I. Batatia, G. Csányi, M. Dzamba, P. Eastman, N. C. Frey, X. Fu, V. Gharakhanyan, A. S. Krishnapriyan, J. A. Rackers, S. Raja, A. Rizvi, A. S. Rosen, Z. Ulissi, S. Vargas, C. L. Zitnick, S. M. Blau, and B. M. Wood, The Open Molecules 2025 (OMol25) Dataset, Evaluations, and Models, arXiv:2505.08762 (2025).
